# Supplementary material for: Dynamic anticipation by Cdk2/Cyclin A-bound p27 mediates signal integration in cell cycle regulation
Source: Nat Commun. 2019 Apr 11;10:1676. doi: 10.1038/s41467-019-09446-w (PMC6459857; doi:10.1038/s41467-019-09446-w)
Supplement: Supplementary file 1 — Supplementary Information [file 41467_2019_9446_MOESM1_ESM.pdf]

Dynamic anticipation by Cdk2/Cyclin A-bound p27 mediates signal integration in cell cycle regulation

M. Tsytlonok, H. Sanabria, Y. Wang *et al.*

Supplementary Information

Supplementary Methods

Burst analysis and parametric lines for FRET and anisotropy multidimensional histograms

To identify single-molecule events we use "burstwise" or "time-window" selection with a count rate that is different by a 2σ criteria out of the mean background count rate. Cutoff times may vary from sample to sample depending on the background signal. Time-resolved fluorescence histograms of each single-molecule event, with a minimum of 60 photons, is processed and fitted using a maximum likelihood algorithm[1] in custom developed programs coded in LabVIEW (National Instruments Co.). Fluorescent bursts are plotted in 2D histograms (Origin 8.6, OriginLab Co).

For single-molecule Förster Resonance Energy Transfer (smFRET) experiments, bursts are shown using a parametric relationship between the ratio of the donor fluorescence over the acceptor fluorescence (F_D/F_A) and the fluorescence-weighted donor lifetime obtained in burst analysis $\langle\tau_{D(A)}\rangle_f$. F_D/F_A depends on specific experimental parameters such as count rate per color channel ($\langle S_G\rangle$ and $\langle S_R\rangle$), the fluorescence quantum yields of the dyes ($\Phi_{FD(0)}$ and Φ_{FA} for donor and acceptor respectively), background ($\langle B_G\rangle$ and $\langle B_R\rangle$ for green and red channels), detection efficiencies (g_G and g_R for green and red respectively) and crosstalk (α) following these relationships

$$F_D = \frac{S_G - \langle B_G\rangle}{g_G}, \tag{1}$$

$$F_A = \frac{S_R - \alpha F_G - \langle B_R\rangle}{g_R}. \tag{2}$$

In the F_D/F_A vs. $\langle\tau_{D(A)}\rangle_f$ 2D representations it is useful to represent a static FRET line that represent the parametric relationship between (F_D/F_A) and $\langle\tau_{D(A)}\rangle_f$ which include the dynamics of the fluorophore's linker. Consequently, the linker flexibility generates a distribution of distances instead of a single distance. Mathematically, the FRET lines for F_D/F_A vs. $\langle\tau_{D(A)}\rangle_f$ and R_{DA} vs. $\langle\tau_{D(A)}\rangle_f$, corrected for linker mobility, are

$$\left(\frac{F_D}{F_A}\right)_{\text{static,L}} = \frac{\Phi_{FD(0)}}{\Phi_{FA}} \cdot \left(\frac{\tau_{D(0)}}{\sum_{i=0}^{3} A_{i,L} \left(\langle\tau_{D(A)}\rangle_{x,L}\right)^i} - 1 \right)^{-1}. \tag{3a}$$

$$R_{DA\text{static,L}} = R_0 \cdot \left(\frac{1}{\frac{\tau_{D(0)}}{\sum_{i=0}^{2} B_{i,L} \left(\langle\tau_{D(A)}\rangle_{x,L}\right)^i} - 1} \right)^{-1/6}. \tag{3b}$$

The L sub index notation is to identify and specify the linker effects and $\tau_{D(0)}$ is the donor fluorescence lifetime in the absence of acceptor. The "$A_{i,L}$" and "$B_{i,L}$" coefficients are empirically determined by a polynomial approximation of the following parametric relationship between the species average lifetime $\langle\tau_{D(A)}\rangle_{x,L}$ and fluorescence weighted average lifetime $\langle\tau_{D(A)}\rangle_{f,L}$ for a range for $\langle R_{DA}\rangle$ =[1 Å to 5 R_0] Å using the following relationships. The determination procedure is found elsewhere.[2]

$$\langle\tau_{D(A)}\rangle_{x,L}=\sum_{i=0}^{3}A_{i,L}\left(\langle\tau_{D(A)}\rangle_{f,L}\right)^{i}$$

$$\langle\tau_{D(A)}\rangle_{f}=\langle\tau_{D(A)}\rangle_{f,L}=\frac{\int\tau_{D(A)}{}^{2}p(R_{DA})dR_{DA}}{\langle\tau_{D(A)}\rangle_{x,L}} \tag{4}$$

$$\langle\tau_{D(A)}\rangle_{x,L}=\int\tau_{D(A)}p(R_{DA})dR_{DA}$$

Here, the distribution of distances is assumed to follow a Gaussian probability function with a mean FRET distance $\langle R_{DA}\rangle$ and standard deviation σ_{DA},

$$p(R_{DA})=\frac{1}{\sqrt{2\pi}\sigma_{DA}}\exp\left(-\frac{\left(R_{DA}-\langle R_{DA}\rangle\right)^{2}}{2\sigma_{DA}^{2}}\right). \tag{5}$$

Keep in mind that in Eq. 4 there is a R_{DA} for each $\tau_{D(A)}$ following the Förster relationship

$$\tau_{D(A)}=\tau_{D(0)}\cdot\left(1+\left(\frac{R_0}{R_{DA}}\right)^{6}\right)^{-1}, \tag{6}$$

where R_0 is the Förster distance. For our particular set of dyes R_0 = 52 Å and we assume isotropic reorientation of dyes (κ^2 = 2/3) in the determination of R_0. The static FRET line corrected for linker dynamics is only valid for cases where there is no dynamic interexchange between states.
In addition to the static FRET line, we use the dynamic FRET line to show the interexchange between states. In this case, a mixed fluorescence species arises from the interconversion between two conformational states, where each state follows the Gaussian distribution stated above. For the simplest case the dynamic FRET line can be presented as[3]

$$\left(\frac{F_D}{F_A}\right)_{\mathrm{dyn,L}}=\frac{\Phi_{FD(0)}}{\Phi_{FA}\tau_{D(0)}}\cdot\frac{\langle\tau_1\rangle_f\cdot\langle\tau_2\rangle_f}{\left(\langle\tau_1\rangle_f+\langle\tau_2\rangle_f-\sum_{i=0}^{3}C_{i,L}\left(\langle\tau_{D(A)}\rangle_f\right)^{i}\right)-\frac{\langle\tau_1\rangle_f\cdot\langle\tau_2\rangle_f}{\tau_{D(0)}}}, \tag{7}$$

where $\langle\tau_{D(A)}\rangle_{f,L}$ is the mixed fluorescence lifetime, and $\langle\tau_1\rangle_f$ and $\langle\tau_2\rangle_f$ are two donor fluorescence lifetimes in presence of acceptor corresponding to the states that give rise to the dynamic exchange. The "$C_{i,L}$" coefficients are determined for each FRET pair and differ from the "$A_{i,L}$" coefficients in the static FRET lines. The process of determining these coefficients can be found elsewhere.[2] The *L* sub index notation is to identify and specify the linker effects. To represent single-molecule Fluorescence Anisotropy (smFA) we chose the scatter-corrected fluorescence anisotropy per burst (r_D)[4],), which is calculated as

$$r_D=\frac{G_r\left(S_{\|}-\langle B_{\|}\rangle\right)-\left(S_{\perp}-\langle B_{\perp}\rangle\right)}{G_r\left(S_{\|}-\langle B_{\|}\rangle\right)(1-3l_2)+\left(S_{\perp}-\langle B_{\perp}\rangle\right)(2-3l_1)}, \tag{8}$$

where G_r is the ratio of the detection efficiencies ($g_{\perp}/g_{||}$) on the perpendicular ($\perp$) and parallel (||) channels, sometimes referred as *G*-factor, l_1 = 0.01758 and l_2 = 0.0526 are correction factors for the depolarization created by the microscope objective,[4, 5] and the signal in the parallel and perpendicular detector are $S_{||}$ and $S_{\perp}$ respectively.

In smFA experiments, the parametric histogram used is r_D vs. $\langle \tau_D \rangle$ which relates the steady state anisotropy and the average fluorescence lifetime per burst. In ensemble conditions there is a similar relation called Perrins' Eq.

$$r_D = r_0 \left(1 + \frac{\langle \tau_D \rangle}{\rho}\right)^{-1}, \qquad (9)$$

where r_0 is the fundamental anisotropy of the fluorophore and is set to be 0.38. ρ is the rotational correlation time and $\langle \tau_D \rangle$ is the average fluorescence lifetime per burst.

Probability Distribution Analysis (PDA) of single-molecule Fluorescence Anisotropy (smFA) and Förster Resonance Energy Transfer (smFRET) experiments

To model the shape of the anisotropy and F_D/F_A distributions, we use probability distribution analysis or PDA. Anisotropy-PDA is an extension of the FRET-PDA theory which was derived first. The theory behind these can be found in [6, 7]. In short, the measured fluorescence signal *S,* consisting of fluorescence (*F*) and background (*B*) photons are expressed in photon count numbers per time window (Δt) of a fixed length. In Multiparameter Fluorescence Detection the signal is split into two spectral windows termed "green" and "red" each with two polarization components (Parallel "||" and Perpendicular "$\perp$"). The probability of observing a certain combination of photon counts in two detection channels 1 and 2 (e.g., "1=green" and "2=red" or "1=||" and "2=$\perp$") and measured by two or more single photon counting detectors., $P(S_1, S_2)$, is given by a product of independent probabilities

$$P(S_1, S_2) = \sum_{F_1+B_1=S_1;\,F_2+B_2=S_2} P(F)P(F_1, F_2 \mid F)P(B_1)P(B_2). \qquad (10)$$

$P(F)$ describes the fluorescence intensity distribution, i.e., the probability of observing exactly F fluorescence photons per time window (Δt). $P(B_1)$ and $P(B_2)$ represent the background intensity distributions. $P(F_1, F_2 \mid F)$ is the conditional probability of observing a particular combination of F_1 and F_2, provided the total number of fluorescence photons is F.This can be expressed as

$$P(F_1, F_2|F) = \frac{F!}{F_1!F_2!} p_1^{F_1} p_2^{F_2} = \frac{F!}{F_1!(F-F_1)!} p_1^{F_1}(1-p_1)^{F-F_1}. \qquad (11)$$

p_1 stands for the probability of a detected photon to be registered by the first detector (e.g., green in a FRET experiment or parallel in an anisotropy experiment). For the case of smFA experiments $p_1 = p_{||}$ and it is written as

$$p_{||} = \frac{1 + \langle r \rangle (2 - 3l_1)}{1 + \langle r \rangle (2 - 3l_1) + G_r - G_r \langle r \rangle (1 - 3l_2)}; \quad p_\perp = 1 - p_{||} \qquad (12)$$

where G_r, l_1 and l_2 were previously defined. Consequently, $p_2 = p_\perp$.

For smFRET p_1 is unambiguously related to the FRET efficiency E according to

$$p_{1|} = \left(1 + \alpha + \frac{E\Phi_{FA}}{(1-E)G\Phi_{FD(0)}}\right) \quad p_2 = 1 - p_{1|}. \qquad (13)$$

Here, G stands for the ratio of the detection efficiencies in the spectral windows ($G = g_G/g_R$) and the quantum yields ($\Phi_{FD(0)}$ and Φ_{FA}) were previously defined.

The distribution $P(F)$ in Eq. (10) is not directly measurable, instead the total signal intensity distribution $P(S)$ is measured, which is given by

$$P(S) = P(F) \otimes P(B) \quad (14)$$

where $P(B)$ is the distribution probability of background counts. Details on the deconvolution procedure are described elsewhere [7]. Finally, Eq (10) can be extended for multiple species with the brightness correction used in this work [8]. Each species distributions has a half width (hw_{DA}) which depends mostly on shot noise and photophysical properties of the acceptor fluorophore.

Statistical uncertainties in PDA

Confidence intervals estimation for multiple fit parameters is performed as follows. All free fit parameters are varied simultaneously in a random manner. The χ_r^2 value is calculated at 100000 random points yielding 100-1000 points with χ_r^2 values below $\chi_{r,\max}^2$

$$\chi_{r,\max}^2 = \chi_{r,\min}^2 + \sqrt{\frac{2}{N}} \quad (15)$$

where N is the number of bins and $\chi_{r,\min}^2$ is the reduced chi-squared value of the best fit. The threshold of $\chi_{r,\max}^2$ is assigned as 1σ confidence interval. One could calculate thresholds. Alternative methods for threshold determination are available [9]; however, in practice $\chi_{r,\min}^2$ is often affected by experimental imperfections and can be considerably larger than one. For this reason, we prefer this test to measure the robustness of the fits providing numerical uncertainties of the free parameters.

Filtered Fluorescence Correlation Spectroscopy

To separate species, we use filtered FCS (fFCS) [10, 11]. fFCS differs from standard FCS [12] and FRET-FCS [13] by interrogating the "species" (conformational states) fluctuations instead of photon count rates [13]. We define the species auto- or cross-correlation function as

$$G^{(i,m)}(t_c) = \frac{\left\langle F^{(i)}(t) \cdot F^{(m)}(t+t_c) \right\rangle}{\left\langle F^{(i)}(t) \right\rangle \cdot \left\langle F^{(m)}(t+t_c) \right\rangle} = \frac{\left\langle \left(\sum_{j=1}^{d \cdot L} f_j^{(i)} \cdot S_j(t) \right) \cdot \left(\sum_{j=1}^{d \cdot L} f_j^{(m)} \cdot S_j(t+t_c) \right) \right\rangle}{\left\langle \sum_{j=1}^{d \cdot L} f_j^{(i)} \cdot S_j(t) \right\rangle \cdot \left\langle \sum_{j=1}^{d \cdot L} f_j^{(m)} \cdot S_j(t+t_c) \right\rangle}, \quad (16)$$

where (i) and (m) are two selected "species" in a mixture. When $i=m$ we say it is the species auto-correlation function (sACF), and when $i \neq m$ it is the species cross-correlation function (sCCF). The difference from standard FCS is that in fFCS we introduce a set of filters, $f_j^{(i)}$, that depend on the arrival time of each photon after each excitation pulse. The signal $S_j(t)$, obtained via pulsed excitation is recorded at each j = 1 ... L TCSPC-channel. The signal and filters per detector, d, are stacked in a single array with dimensions $d \cdot L$ for global minimization as previously shown [10]. Filters are defined in such a way that the relative "error" difference between the photon count per species ($w^{(i)}$) and the weighted histogram $f_j^{(i)} \cdot H_j$ is minimized as defined in Eq. (17).

$$\left\langle \left(\sum_{j=1}^{d\cdot L} f_j^{(i)} \cdot H_j - w^{(i)} \right)^2 \right\rangle \to \min. \qquad (17)$$

where brackets represent time averaging.

The requirement is that the decay histogram H_j can be expressed as a linear combination of the conditional probability distributions $p_j^{(i)}$, such as $H_j = \sum_{i=1}^{n} w^{(i)} p_j^{(i)}$, with $\sum_{j=1}^{d\cdot L} p_j^{(i)} = 1$. Hence, the sCCF provides maximal contrast for intercrossing dynamics [10]. One major advantage of sCCF is that if photophysical properties are decoupled from species selection the intercrossing dynamics [13] is recovered with great fidelity.

To properly fit the species auto- and cross-correlation function we used a set of equations previously presented [10]

$$G_{i,i}(t_c) = 1 + \frac{1}{N_{Br}} \cdot G_{diff}^{(i)}(t_c) \cdot \left[1 - T^{(i)} + T^{(i)} \cdot \mathbf{exp}\left(-t_c/t_T^i\right) + \sum_{R=1}^{4} AC_{i,i}^{(R)} \cdot \left(\mathbf{exp}\left(-\frac{t_c}{t_R}\right) - 1 \right) \right] \cdot G_B^{(i)}(t_c)$$

$$G_{m,m}(t_c) = 1 + \frac{1}{N_{Br}} \cdot G_{diff}^{(m)}(t_c) \cdot \left[1 - T^{(m)} + T^{(m)} \cdot \mathbf{exp}\left(-t_c/t_T^m\right) + \sum_{R=1}^{4} AC_{m,m}^{(R)} \cdot \left(\mathbf{exp}\left(-\frac{t_c}{t_R}\right) - 1 \right) \right] \cdot G_B^{(m)}(t_c)$$

$$G_{i,m}(t_c) = 1 + \frac{1}{N_{CC}} \cdot G_{diff}^{(i,m)}(t_c) \cdot \left[1 - CC_{i,m} \cdot \sum_{R=1}^{4} X_{i,m}^{(R)} \cdot \mathbf{exp}\left(-\frac{t_c}{t_R}\right) \right] \cdot \left(1 - B_{i,m} \cdot \mathbf{exp}\left(-\frac{t_c}{t_B}\right) \right)$$

(18)

where t_R are the relaxation times that correspond to the exchange times between selected species with corresponding absolute amplitudes of the sACF $AC_{x,x}^{(R)}$ and the relative normalized amplitudes of the sCCF $CC_{x,x}^{(R)}$. $T^{(x)}$ is the triplet amplitude, however, triplet states dynamic was not found in the measured samples. N_{br} is the number of bright molecules in the sACF's in the focus and N_{CC} of the sCCF's corresponds to the inverse of the initial amplitude $G_{i,m}(0)$. $G_B^{(x)}(t_c)$ is defined for bleaching term:

$$G_B^{(x)}(t_c) = 1 - B^{(x)} + B^{(x)} \cdot \exp\left(-t_c/t_B\right), \qquad (19)$$

$G_{diff}^{(x)}(t_c)$ is the diffusion term of species *x*:

$$G_{diff}^{(x)}(t_c) = \left(1 + \frac{t_c}{t_{diff}^{(x)}}\right)^{-1} \cdot \left(1 + \left(\frac{\omega_0}{z_0}\right)^2 \cdot \frac{t_c}{t_{diff}^{(x)}}\right)^{-\frac{1}{2}}. \qquad (20)$$

A 3-dimensional Gaussian shaped volume element parameters ω_0 and z_0 is considered. We assume that $G_{diff}(t_c) = G_{diff}^{(i)}(t_c) = G_{diff}^{(m)}(t_c)$ take the form of Eq. (20).In fFCS the amplitudes are highly dependent on

the brightness of the individual sub-states and the exchange rate constants. Thus a direct interpretation is not straightforward [14].

Accessible volume (AV) model and inter-fluorophore distances

To accurately compare FRET-derived distances with structural information provided by crystallography data it is imperative to consider the dimensions of the fluorophores. To do so, we compute the accessible volume of the dyes by considering them as hard sphere models connected to the protein via flexible linkers (modeled as a flexible cylindrical pipe) [15]. The overall dimension (width and length) of the linker is based on their chemical structures. For Alexa 488 maleimide the five carbon linker length was set to 20 Å, the width of the linker is 4.5 Å and a three sphere model was used to model the dye R_1= 5.0 Å, R_2= 4.5 Å and R_3= 1.5 Å. For Alexa 647 maleimide the dimensions used were: length = 22 Å, width = 4.5 Å and the dye radii R_1= 11.0 Å, R_2= 4.7 Å and R_3= 1.5 Å.

To account for dye linker mobility we generated a series of AV's for donor and acceptor dyes attached to p27 placing the dyes at multiple separation distances. For each pair of AV's, we calculated the distance between dye mean positions (R_{mp})

$$R_{mp} = \left| \left\langle \vec{R}_{D(i)} \right\rangle - \left\langle \vec{R}_{A(j)} \right\rangle \right| = \left| \frac{1}{n} \sum_{i=1}^{n} \vec{R}_{D(i)} - \frac{1}{m} \sum_{j=1}^{m} \vec{R}_{A(j)} \right|, \quad (21)$$

where $\vec{R}_{D(i)}$ and $\vec{R}_{A(i)}$ are all the possible positions that the donor fluorophore and the acceptor fluorophore can take. However, in single-molecule FRET experiment where a ratiometric FRET is calculated the distances is weighted by the average fluorescence thus the mean donor-acceptor distance observed is

$$\left\langle R_{DA} \right\rangle_E = R_0 \left(\left\langle E \right\rangle^{-1} - 1 \right)^{1/6} \quad (22)$$

where the average efficiency is defined as $\left\langle E \right\rangle = \frac{1}{nm} \sum_{i=1}^{n} \sum_{j=1}^{m} \left(\frac{R_0}{R_0 + \left| \overrightarrow{R_D^{(i)}} - \overrightarrow{R_A^{(j)}} \right|^6} \right)$.

Stoichiometric phosphorylation of p27 and p27-KID for single-molecule fluorescence studies

For phosphorylating p27 with either Src-KD or SRC-KD plus Abl-KD, the protocol described earlier was used.[16] We have gone at great length to ensure stoichiometric phosphorylation and the absence of non-phosphorylated protein that could interfere with the experiments. First, we have checked the kinetics of full length p27 phosphorylation (reaction carried out as in Supplementary Figure 1 and 2 with full length p27) and found that it reached saturation and p27 could not be further phosphorylated upon removing kinase and adding fresh enzyme (Supplementary Figure 14A). In the case of p27-KID, phosphorylation by Src-KD causes a shift in the position of the band, and the absence of the protein at the original position shows that there is no non-phosphorylated material left (Supplementary Figure 14B). Second, for several p27 variants labelled with BODIPY-FL (p27-C29, -C54 and -C93)), we have bound phosphorylated protein to a specific phospho-protein binding membrane of the Pro-Q® Diamond Phosphoprotein Enrichment Kit (Invitrogen), applied extensive washing to ensure that all non-phosphorylated protein is removed (due to

which about 90% of the phosphorylated protein was also lost). As shown by a Western blot stained by Phospho-Tyrosine Mouse mAb (Bioke), the remaining 10% was at least 98% phosphorylated (Supplementary Figure 14C). The remaining amount was still more than enough for the single-molecule fluorescence measurements. The homogeneity of phosphorylated p27 vs. non-phosphorylated was also confirmed by the single-molecule fluorescence experiments, in which the phosphorylated and (very little) non-phosphorylated p27 could be seen separately.

Supplementary Tables

Supplementary Table 1. Data collection and refinement statistics for determination of the structure of the Cdk2/cyclin A/p27-KID-ΔC complex using X-ray crystallography.

The structure of the Cdk2/cyclin A/p27-KID-ΔC complex has been deposited and validated bythe PDB with the file name 6ATH for release upon publication.

	**Cdk2/cyclin A/p27-KID-ΔC**
**Data Collection** [a]	
Space Group	$P2_12_12_1$
Cell dimensions	
a,b,c (Å)	74.2, 77.6, 137.5
α, β, γ (°)	90.0, 90.0, 90.0
No. of crystals	2
Wavelength (Å)	1.0
Resolution (Å)	50.0-1.82 (1.89-1.82)
No. unique reflections	71,452 (6,848)
R_{merge} [b]	0.085 (0.522)
Completeness (%)	99.2 (96.5)
Redundancy	10.7 (4.2)
I/σ	26.6 (2.1)
**Refinement**	
Resolution (Å)	29.6-1.82
No. of reflections	71,378
R_{work}/R_{free} [c]	0.171/0.187
No. atoms	
Protein	4,774
Ion	5
Water	399
Average B-factor (Å^2)	28.0
R.m.s. deviations	
Bond lengths (Å)	0.005
Bond angles (°)	0.94
Ramachandran plot	
Favored (%)	98.1
Allowed (%)	1.4
Outliers (%)	0.5

[a] Values in parenthesis are for highest-resolution shell.

[b] $R_{merge} = \sum |I - \langle I \rangle| / \sum I$, where I is the observed intensity.

[c] R_{free} is the R value obtained for a test set of reflections consisting of randomly selected 5% subset of the data set excluded from refinement.

Supplementary Table 2. Fit parameters of smFA experiments obtained by PDA (Sections S1.2, S1.3)**.**

A) 2-state model fit parameters

Residue number	Anisotropy (Fraction (%)) No phosphorylation		χ_r^2	Anisotropy (Fraction (%)) pY88		χ_r^2	Anisotropy (Fraction (%)) pY74/pY88		χ_r^2
	Low r_D	High r_D		Low r_D	High r_D		Low r_D	High r_D	
29	0.08±0.01 (12.9±1.9)	0.26±0.01 (87.1±13.1)	1.26	0.07±0.01 (25.6±3.1)	0.26±0.01 (74.4±8.9)	1.37	0.06±0.01 (26.7±3.5)	0.25±0.01 (73.3±9.7)	1.39
40	0.13±0.01 (57.7±7.6)	0.25±0.01 (42.3±5.5)	1.54	0.12±0.01 (62.0±5.8)	0.24±0.01 (38.0±3.6)	2.06	0.13±0.01 (61.8±7.0)	0.25±0.01 (38.2±4.3)	1.82
54	0.04±0.01 (30.8±5.7)	0.24±0.01 (69.2±12.9)	1.45	0.08±0.01 (29.9±4.9)	0.24±0.01 (70.1±11.6)	1.29	0.07±0.01 (71.7±6.8)	0.23±0.01 (28.3±2.7)	1.14
75	0.07±0.01 (27.2±4.8)	0.20±0.01 (72.8±13)	1.26	0.09±0.01 (25.4±3.5)	0.26±0.01 (74.6±10.1)	1.67	0.11±0.01 (40.5±4.6)	0.26±0.01 (59.5±6.5)	1.97
93	0.08±0.01 (33.3±5.1)	0.28±0.01 (66.7±10.1)	1.18	0.11±0.02 (65.9±12.3)	0.27±0.02 (34.1±6.3)	1.32	0.09±0.01 (65.1±10.4)	0.25±0.01 (34.9±5.6)	1.28

B) Comparative fit statistics between 1-, 2-, and 3-state model for smFA with BODIPY labels.

BODIPY Samples (**No P**) In complex Cdk2/Cyclin A	χ_r^2				
	1 state	2 states*	3 states	$\lvert \Delta\chi^2_{r,1-2} \rvert$	$\lvert \Delta\chi^2_{r,3-2} \rvert$
p27 C29	17.40	1.26	0.99	16.1	0.27
p27 C40	16.70	1.54	1.24	15.2	0.30
p27 C54	13.50	1.45	1.05	12.1	0.40
p27 C75	8.74	1.26	0.97	7.5	0.29
p27 C93	20.50	1.18	0.91	19.3	0.27
* χ_r^2 as reported in Table S2A					

Supplementary Table 3. Brightness correction for smFA experiments.
Normalized to the maximum lifetime: $Q^{(\text{short lifetime})} = \tau_D^{(\text{short})}/\tau_D^{(\text{long})}$.

Sample in complex	$\tau_D^{(\text{High rD})}$ [ns]	$\tau_D^{(\text{Low rD})}$ [ns]	$Q^{(\text{High rD}}$	$Q^{(\text{Low rD})}$
p27 C29 No phosphorylation	4.6	5.4	0.85	1
pY88-p27 C29	4.6	5.4	0.85	1
pY74/pY88-p27 C29	4.6	5.4	0.85	1
p27 C40 No phosphorylation	5.9	4.6	1	0.78
pY88-p27 C40	5.9	4.6	1	0.78
pY74/pY88-p27 C40	5.9	4.6	1	0.78
p27 C54 No phosphorylation	5.9	4.2	1	0.71
pY88-p27 C54	5.9	4.2	1	0.71
pY74/pY88-p27 C54	5.9	4.2	1	0.71
p27 C75 No phosphorylation	5.9	4.2	1	0.71
pY88-p27 C75	5.9	4.2	1	0.71
pY74/pY88-p27 C75	5.9	4.2	1	0.71
p27 C93 No phosphorylation	5.9	4.6	1	0.78
pY88-p27 C93	5.9	4.6	1	0.78
pY74/pY88-p27 C93	5.9	4.6	1	0.78

Supplementary Table 4. Rotational correlation time for smFA experiments with BODIPY.

A) Comparing the average anisotropy ($\langle r_D \rangle = b_1 r_{D,Low} + b_2 r_{D,High}$), there is a significant increase of $\langle r_D \rangle$ when p27 is bound to the complex. $\langle r_D \rangle$ reflects the overall tumbling of the observed molecule. The larger $\langle r_D \rangle$ is the larger the molecule, thus reflecting the larger size in the complex form.

Samples<br>**No phosphorylation**	Free/uncomplex<br>$\langle r_D \rangle$	Cdk2/Cyclin A<br>$\langle r_D \rangle$
p27 C29	0.07	0.24
p27 C40	0.09	0.18
p27 C54	0.09	0.18
p27 C75	0.10	0.16
p27 C93	0.17	0.21
Average	0.10	0.19
St. dev.	0.04	0.03

B) Values were obtained using Table 2, Table 3 and Perrin's Equation [Eq. (9)] for Cdk2/cyclin A/p27 samples.

Rotational Correlation (ns)	**No phosphorylation**		**pY88**		**pY74/pY88**	
Residue No.	Low r_D	High r_D	Low r_D	High r_D	Low r_D	High r_D
29	1.5	10.5	1.2	10.5	1.0	9.3
40	2.5	11.9	2.2	10.6	2.5	11.9
54	0.5	10.6	1.1	10.6	1.0	9.4
75	1.0	6.8	1.3	13.5	1.8	13.5
93	1.3	17.6	1.9	15.3	1.5	11.9

Supplementary Table 5. Fluorophore properties of dyes used in smFRET experiments and generated FRET lines according to S1.1 equations (3) and (7).

Sample in complex	$\Phi_{D(0)}$	Φ_A	*Static FRET Line*	*Dynamic FRET Line*
p27 C29/54 No phosphorylation	0.70	0.39	$(0.6988/0.39)/((3.7494/((-0.0519*\langle\tau_{D(A)}\rangle_f^3)+(0.3131*\langle\tau_{D(A)}\rangle_f^2)+0.5690*\langle\tau_{D(A)}\rangle_f+-0.0529))-1)$	$0.1913/(0.39*((1/2.044+1/1.264-(1.4769*\langle\tau_{D(A)}\rangle_f+-1.0373)/(1.2640*2.0440))-1/3.7494))$
pY88-p27 C29/54	0.72	0.36	$(0.7220/0.36)/((3.9015/((-0.0470*\langle\tau_{D(A)}\rangle_f^3)+(0.3002*\langle\tau_{D(A)}\rangle_f^2)+0.5507*\langle\tau_{D(A)}\rangle_f+-0.0505))-1)$	$0.1957/(0.36*((1/2.122+1/1.1340-(1.5937*\langle\tau_{D(A)}\rangle_f+-1.3355)/(1.1340*2.122))-1/3.9015))$
pY74/pY88-p27 C29/54	0.72	0.37	$(0.7239/0.37)/((4.0239/((-0.0483*\langle\tau_{D(A)}\rangle_f^3)+(0.2978*\langle\tau_{D(A)}\rangle_f^2)+0.5875*\langle\tau_{D(A)}\rangle_f+-0.0564))-1)$	$0.1872/(0.37*((1/2.04+1/1.06-(1.5244*\langle\tau_{D(A)}\rangle_f+-1.1138)/(1.06*2.04))-1/4.0239))$
E88-p27 C29/54	0.75	0.39	$(0.6036/0.37)/((3.511/((-0.0574*\langle\tau_{D(A)}\rangle_f^3)+(0.2965*\langle\tau_{D(A)}\rangle_f^2)+0.6838*x+-0.0638))-1)$	
E74/E88-p27 C29/54	0.60	0.37	$(0.5948/0.394)/((3.5383/((-0.0556*\langle\tau_{D(A)}\rangle_f^3)+(0.282*\langle\tau_{D(A)}\rangle_f^2)+0.7161*\langle\tau_{D(A)}\rangle_f+-0.067))-1)$	
p27 C54/93 No phosphorylation	0.72	0.42	$(0.7194/0.42)/((3.8301/((-0.0501*\langle\tau_{D(A)}\rangle_f^3)+(0.3112*\langle\tau_{D(A)}\rangle_f^2)+0.5553*\langle\tau_{D(A)}\rangle_f+-0.0513))-1)$	$0.1884/(0.42*((1/2.05+1/1.46-(1.3896*\langle\tau_{D(A)}\rangle_f+-0.8588)/(1.46*2.05))-1/3.8301))$
pY88-p27 C54/93	0.75	0.37	$(0.7519/0.37)/((3.9997/((-0.0443*\langle\tau_{D(A)}\rangle_f^3)+(0.2947*\langle\tau_{D(A)}\rangle_f^2)+0.5367*\langle\tau_{D(A)}\rangle_f+-0.0490))-1)$	$0.1925/(0.37*((1/2.14+1/1.49-(1.4414*\langle\tau_{D(A)}\rangle_f+-1.029)/(1.49*2.14))-1/3.9997))$
pY74/pY88-p27 C54/93	0.75	0.36	$(0.7541/0.36)/((3.9983/((-0.0468*\langle\tau_{D(A)}\rangle_f^3)+(0.3068*\langle\tau_{D(A)}\rangle_f^2)+0.5289*\langle\tau_{D(A)}\rangle_f+-0.0476))-1)$	$0.1943/(0.36*((1/2.14+1/1.49-(1.4602*\langle\tau_{D(A)}\rangle_f+-1.0788)/(1.49*2.14))-1/3.9983))$
E88-p27 C54/93	0.63	0.33	$(0.6036/0.363)/((3.511/((-0.0574*\langle\tau_{D(A)}\rangle_f^3)+(0.2965*\langle\tau_{D(A)}\rangle_f^2)+0.6838*\langle\tau_{D(A)}\rangle_f+-0.0638))-1)$	
E74/E88-p27 C54/93	0.67	0.36	$(0.683/0.33)/((3.7855/((-0.0475*\langle\tau_{D(A)}\rangle_f^3)+(0.2846*\langle\tau_{D(A)}\rangle_f^2)+0.6179*\langle\tau_{D(A)}\rangle_f+-0.0575))-1)$	
p27 C75/110 No phosphorylation	0.63	0.39	$(0.6324/0.39)/((3.7203/((-0.0510*\langle\tau_{D(A)}\rangle_f^3)+(0.2782*\langle\tau_{D(A)}\rangle_f^2)+0.6777*\langle\tau_{D(A)}\rangle_f+-0.0646))-1)$	$0.175/(0.39*((1/2.13+1/1.02-(1.3731*\langle\tau_{D(A)}\rangle_f+-0.7835)/(1.02*2.13))-1/3.7203))$
pY88-p27 C75/110	0.63	0.36	$(0.6332/0.36)/((3.7901/((-0.0477*\langle\tau_{D(A)}\rangle_f^3)+(0.2612*\langle\tau_{D(A)}\rangle_f^2)+0.7019*\langle\tau_{D(A)}\rangle_f+-0.0666))-1)$	$0.1681/(0.36*((1/2.11+1/1.56-(1.1605*\langle\tau_{D(A)}\rangle_f+-0.2981)/(1.56*2.11))-1/3.5651))$

pY74/pY88-p27 C75/110	0.60	0.37	$\frac{(0.6025/0.37)/((3.7301/((-0.0486*\langle\tau_{D(A)}\rangle_f^3)+(0.2502*\langle\tau_{D(A)}\rangle_f^2)+0.7510*\langle\tau_{D(A)}\rangle_f+-0.0711))-1)}{0.1624/(0.37*((1/1.77+1/1.32-(1.1961*\langle\tau_{D(A)}\rangle_f+-0.3101)/(1.3200*1.77))-1/3.7301))}$
E88-p27 C75/110	0.67	0.38	$\frac{(0.7129/0.385)/((3.8577/((-0.0456*\langle\tau_{D(A)}\rangle_f^3)+(0.2868*\langle\tau_{D(A)}\rangle_f^2)+0.5847*\langle\tau_{D(A)}\rangle_f+-0.0539))-1)}{}$
E74/E88-p27 C75/110	0.71	0.39	$\frac{(0.6482/0.375)/((3.7123/((-0.0497*\langle\tau_{D(A)}\rangle_f^3)+(0.2802*\langle\tau_{D(A)}\rangle_f^2)+0.6602*\langle\tau_{D(A)}\rangle_f+-0.0616))-1)}{}$

Supplementary Table 6. Fit parameters of PDA analysis for smFRET experiments.

Global fitting ΔT=1, 2 and 3 ms. χ^2_r is shown for ΔT=3 ms. A) All fractions B) Renormalized fractions for only FRET species. Half-width distribution (hw_{DA}) of the PDA distribution should not be confused with σ_{DA} in Eq. (5). Data was corrected as described in S1.2 and using values from Table 3. C) Comparative fit statistics between 1, 2, and 3-state model for FRET samples.

**A)** Samples Cdk2/cyclin A/p27	$\langle R_{DA}\rangle_E$ [Å] (Fraction (%)) hw_{DA} [Å] **No P**				χ_r^2	$\langle R_{DA}\rangle_E$ [Å] (Fraction (%)) hw_{DA} [Å] **pY88**				χ_r^2	$\langle R_{DA}\rangle_E$ [Å] (Fraction (%)) hw_{DA} [Å] **pY74/pY88**				χ_r^2
	Low $\langle R_{DA}\rangle_E$	High $\langle R_{DA}\rangle_E$	Dirt	D_{only}		Low $\langle R_{DA}\rangle_E$	High $\langle R_{DA}\rangle_E$	Dirt	D_{only}		Low $\langle R_{DA}\rangle_E$	High $\langle R_{DA}\rangle_E$	Dirt	D_{only}	
	43.1±0.1	52.3±0.2	74.9	10^6		41.6±0.1	52.5±0.1	83.6	10^6		40.9±0.3	52.0±0.3	79.5	10^6	
C29-54	(11.1±0.5)	(59.9±2.7)	(22.8)	(6.2)	4.8	(9.2±0.2)	(30.0±0.6)	(48.6)	(12.2)	37.4	(14.4±1.4)	(22.2±2.1)	(44.3)	(19.1)	5.2
	4.6	2.7	17.7	--		3.3	2.8	18.8	--		2.8	2.5	17.6	--	
	45.2±0.3	52.3±0.3	76.4	10^6		45.2±0.4	52.3±0.4	82.9	10^6		45.2±0.2	52.3±0.2	82.9	10^6	
C54-93	(55.7±2.8)	(11.6±3.2)	(24.7)	(8.0)	5.3	(20.2±1.7)	(16.9 ±2.2)	(52.9)	(10.0)	2.1	(18.9±1.4)	(7.4±0.5)	(44.7)	(29.0)	10.1
	3.0	3.5	16	--		3.7	3.0	15.9	--		3.6	2.0	21.2	--	
	40.7±0.2	54.6±0.3	81.5	10^6		46.8±0.1	54.4±0.1	83.5	10^6		44.3±0.3	49.9±0.4	88.2	10^6	
C75-110	(6.4±0.5)	(37.1±2.7)	(43.1)	(13.5)	5.1	(17.8±0.3)	(13.2±0.2)	(63.7)	(5.2)	71.0	(23.3±3.0)	(15.4±2.0)	(37.5)	(23.9)	3.1
	3.4	3.8	16.6	--		4.4	5.9	16.1	--		2.6	6.2	18.1	--	

**B)** Samples Cdk2/cyclin A/p27	**No P** Fraction (%)		**pY88** Fraction (%)		**pY74/pY88** Fraction (%)	
	Low $\langle R_{DA}\rangle_E$	High $\langle R_{DA}\rangle_E$	Low $\langle R_{DA}\rangle_E$	High $\langle R_{DA}\rangle_E$	Low $\langle R_{DA}\rangle_E$	High $\langle R_{DA}\rangle_E$
C29-54	15.6	84.4	23.5	76.5	39.3	60.7
C54-93	82.8	17.2	54.4	45.6	71.9	28.1
C75-110	14.7	85.3	57.4	42.6	60.2	39.8

**C)** FRET Samples (**No P**) In complex Cdk2/Cyclin A	χ_r^2		
	2 states*	3 states	$\lvert \Delta\chi^2_{r,3-2} \rvert$
p27 C29-54	4.8	4.16	0.64
p27 C54-93	5.3	2.94	2.36
p27 C75-100	5.1	4.19	0.91

* χ_r^2 as reported on Table 6A

Supplementary Table 7. F_D/F_A levels for High and Low $\langle R_{DA}\rangle_E$.
$\langle R_{DA}\rangle_E$ were converted to FD/FA with Eq. (6) and (3) (Table 6).

Sample in ternary complex	F_D/F_A ($\langle R_{DA}\rangle_E^{(Low)}$)	F_D/F_A ($\langle R_{DA}\rangle_E^{(High)}$)
p27 C29/54 No P	0.7	2.02
pY88-p27 C29/54	0.61	2.33
pY74/pY88-p27 C29/54	0.52	2.04
E88-p27 C29/54		
E74/E88-p27 C29/54		
p27 C54/93 No P	0.84	1.85
pY88-p27 C54/93	0.96	2.18
pY74/pY88-p27 C54/93	0.99	2.27
E88-p27 C54/93		
E74/E88-p27 C54/93		
p27 C75/110 No P	0.47	2.2
pY88-p27 C75/110	1.1	2.24
pY74/pY88-p27 C75/110	1.1	1.42
E88-p27 C75/110	0.78	1.42
E74/E88-p27 C75/110		

Supplementary Table 8. Inter-fluorophore distances of p27 FRET variants in complex with Cdk2/cyclin A based on accessible volume calculations (Section S1.5) and comparison with the "major" state in the non-phosphorylated state.

Sample	1JSU [Å]		Cdk2/cyclin A/p27 Fig 1. pdb:1JSU [Å]		Cdk2/cyclin A/p27-ΔC [Å]		Experiment [Å]
	$\langle R_{DA}\rangle_E$	Cα-Cα	$\langle R_{DA}\rangle_E$	Cα-Cα	$\langle R_{DA}\rangle_E$	Cα-Cα	$\langle R_{DA}\rangle_E$
C29-54	53.7	39.3	52.2	39.6	54.3	40.4	52.3
C54-93	49.5	29.8	48.4	30.3	-	-	45.2
C75-110	-	-	55.3	54.1	-	-	54.6

Supplementary Table 9. Characteristic diffusion time determined by Fluorescence Correlation Spectroscopy.

Samples (**No P**)	Free/uncomplex<br>t_{diff} [ms]	Cdk2/Cyclin A<br>t_{diff} [ms]
p27 C29	1.08	1.61
p27 C40	1.27	1.63
p27 C54	0.92	1.58
p27 C75	1.14	1.75
p27 C93	1.48	1.50
Average	1.18	1.61
St. dev.	0.21	0.09
Samples (**No P**)	free	Cdk2/Cyclin A
p27 C54 C29	1.37	1.76
p27 C54 C93	1.35	1.77
p27 C75 C110	1.26	1.52
Average	1.33	1.68
St. dev	0.06	0.14

Supplementary Table 10. Relaxation rate constants obtained by fFCS for Figure 5C in main text (See equations (18-20)).

Correlation		b	N_{CC}/N_{Br}	t_{diff} [ms]	ω_0/z_0	CC	t_{R1} [µs]	X_1/AC_1	t_{R2} [µs]	X_2/AC_2	t_{R3} [µs]	X_3/AC_3	t_{R4} [µs]	X_4/AC_4	B	t_B [ms]
**p27 C29 C54 in complex with Cdk2/cyclin A**																
No P	LF-HF	1.01	0.37	1.76	3.70	1.24	0.058	0.52	1.96	0.17	22.7	0.24	207	0.07	0.10	4.03
	HF-LF	1.01	0.29			1.27									0.30	
	LF-LF	1.01	0.049					0.35		0.078		0.066		0.047	0.10	
	HF-HF	1.00	0.060					0.37		0.062		0.10		0.023	0.00	
pY88	LF-HF	1.00	0.77	1.69	4.87	2.05	0.058	0.49	1.77	0.16	21.6	0.22	349	0.13	0.16	2.98
	HF-LF	1.00	0.58			2.21									0.38	
	LF-LF	1.00	0.066					0.16		0.066		0.087		0.18	0.29	
	HF-HF	1.00	0.051					0.33		0.083		0.10		0.049	0.15	
pY88-pY74	LF-HF	1.00	0.81	1.86	3.95	2.82	0.058	0.50	1.77	0.14	20.5	0.23	355	0.13	0.25	2.43
	HF-LF	1.01	0.57			2.75									0.47	
	LF-LF	1.00	0.040					0.19		0.060		0.079		0.19	0.34	
	HF-HF	0.99	0.043					0.30		0.097		0.089		0.067	0.24	
**p27 C54 C93 in complex with Cdk2/cyclin A**																
No P	LF-HF	1.00	0.34	1.77	3.42	0.82	0.058	0.50	1.39	0.12	18.3	0.29	247	0.09	0.01	5.76
	HF-LF	1.02	0.20			0.89									0.44	
	LF-LF	1.01	0.039					0.30		0.075		0.086		0.084	0.10	
	HF-HF	1.01	0.041					0.35		0.065		0.096		0.033	0.05	
pY88	LF-HF	1.00	0.78	1.48	4.83	1.45	0.058	0.66	1.27	0.15	25.1	0.14	345	0.05	0.00	3.94
	HF-LF	1.00	0.41			1.55									0.50	
	LF-LF	1.00	0.061					0.17		0.091		0.074		0.12	0.17	
	HF-HF	0.99	0.052					0.38		0.093		0.088		0.022	0.12	
pY88-pY74	LF-HF	1.00	1.12	1.38	4.27	3.16	0.058	0.62	2.22	0.16	27.7	0.13	351	0.09	0.00	3.70
	HF-LF	1.01	0.44			3.64									0.66	
	LF-LF	1.00	0.034					0.20		0.078		0.074		0.16	0.19	

	HF-HF	1.00	0.048					0.37		0.087		0.080		0.035	0.11	
**p27 C75 C110 in complex with Cdk2/cyclin A**																
No P	LF-HF	1.00	0.69	1.52	4.05	2.30	0.043	0.77	1.06	0.14	15.1	0.05	188	0.04	0.00	2.53
	HF-LF	1.01	0.35			0.96									0.35	
	LF-LF	1.01	0.076					0.17		0.072		0.11		0.10	0.03	
	HF-HF	1.00	0.053					0.39		0.12		0.088		0.049	0.10	
pY88	LF-HF	1.00	1.42	1.31	3.99	2.74	0.032	0.72	1.78	0.17	18.2	0.08	195	0.03	0.01	2.76
	HF-LF	1.00	0.47			1.85									0.56	
	LF-LF	1.01	0.090					0.13		0.079		0.092		0.086	0.00	
	HF-HF	1.00	0.062					0.23		0.095		0.088		0.025	0.13	
pY88-pY74	LF-HF	1.00	1.01	1.45	5.57	1.91	0.094	0.63	1.35	0.19	27.2	0.15	286	0.03	0.00	3.60
	HF-LF	1.01	0.34			1.47									0.55	
	LF-LF	1.00	0.049					0.23		0.028		0.065		0.080	0.13	
	HF-HF	0.99	0.050					0.16		0.21		0.093		0.025	0.087	

Supplementary Table 11. Result of the analysis of triplicate measurements of p27-KID, pY88-p27-KID, and pY74/Y88-p27-KID binding to Cdk2/cyclin A, and separately to cyclin A or Cdk2, using surface plasmon resonance (SPR).

The data were fit to a 1:1 Langmuir interaction model.

Interacting species	k_a ($M^{-1}s^{-1}$)	k_d (s^{-1})	K_D (nM)	Rmax (RU)
p27-KID + Cdk2/cyclin A	$1.52 \pm 0.01 \times 10^6$	$5.0 \pm 0.2 \times 10^{-4}$	0.33 ± 0.01	32.2 ± 0.1
pY88-p27-KID + Cdk2/cyclin A	$1.63 \pm 0.01 \times 10^6$	$9.8 \pm 0.1 \times 10^{-4}$	0.60 ± 0.01	17.4 ± 0.1
pY74/Y88-p27-KID + Cdk2/cyclin A	$1.64 \pm 0.01 \times 10^6$	$4.80 \pm 0.03 \times 10^{-3}$	2.93 ± 0.02	34.8 ± 0.1
p27-KID + cyclin A	$4.8 \pm 0.1 \times 10^6$	$1.36 \pm 0.03 \times 10^{-1}$	28 ± 1	21.7 ± 0.2
pY88-p27-KID + cyclin A	$3.93 \pm 0.08 \times 10^6$	$1.46 \pm 0.03 \times 10^{-1}$	37 ± 1	11.0 ± 0.1
pY74/Y88-p27-KID + cyclin A	$2.91 \pm 0.05 \times 10^6$	$1.51 \pm 0.02 \times 10^{-1}$	52 ± 1	22.9 ± 0.2
p27-KID + Cdk2	$1.17 \pm 0.01 \times 10^4$	$4.4 \pm 0.1 \times 10^{-4}$	37.4 ± 0.09	26.9 ± 0.1
pY88-p27-KID + Cdk2	$1.18 \pm 0.01 \times 10^4$	$8.6 \pm 0.1 \times 10^{-4}$	73 ± 1	14.3 ± 0.1
pY74/Y88-p27-KID + Cdk2	$9.28 \pm 0.02 \times 10^3$	$3.37 \pm 0.01 \times 10^{-3}$	363 ± 1	29.0 ± 0.1

Supplementary Table 12. Primers used in cloning and mutagenesis.

	Sequence (5'-3')
p27 22-104 F	CATATGCACCCCAAGCCCTCGGCC
p27 22-104 R	GTCGACTTACTGCGCCGGCACCTTGCAG
p27 1-198 F	CATATGTCAAACGTGCGAGTGTCTAACG
p27 1-198 R	GTCGACTTACGTTTGACGTCTTCTGAGGCCAG
Y89F F	CCGAGTTCTACTTCAGACCCCCG
Y89F R	CGGGGGTCTGAAGTAGAACTCGG
Y88E F	CTTGCCCGAGTTCGAATTCAGACCCCC
Y88E R	GGGGGTCTGAATTCGAACTCGGGCAAG
Y74E F	CTAGAGGGCAAGGAAGAGTGGCAAGAGG
Y74E R	CCTCTTGCCACTCTTCCTTGCCCTCTAG
C29A F	GCCCTCGGCCGCCAGGAACCTC
C29A R	GAGGTTCCTGGCGGCCGAGGGC
C49A F	CTTGGAGAAGCACGCCAGAGACATGGAAG
C49A R	CTTCCATGTCTCTGGCGTGCTTCTCCAAG
C99S F	CCAAAGGTGCCAGCAAGGTGCC
C99S R	GGCACCTTGCTGGCACCTTTGG
C148S F	GTTAGCGGAGCAAAGCGCAGGAATAAG
C148S R	CTTATTCCTGCGCTTTGCTCCGCTAAC
E40C F	GGTGGACCACGAATGCTTAACCCGGGAC
E40C R	GTCCCGGGTTAAGCATTCGTGGTCCACC
E54C F	CAGAGACATGGAATGCGCGAGCCAGCG
E54C R	CGCTGGCTCGCGCATTCCATGTCTCTG
E75C F	CTAGAGGGCAAGTACTGCTGGCAAGAGGTG
E75C R	CACCTCTTGCCAGCAGTACTTGCCCTCTAG
R93C F	CAGACCCCCGTGCCCCCCCAAAG
R93C R	CTTTGGGGGGGCACGGGGGTCTG
S110C F	GCCAGGATGTCTGCGGGAGCC
S110C R	GGCTCCCGCAGACATCCTGGC

Supplementary Figures

A

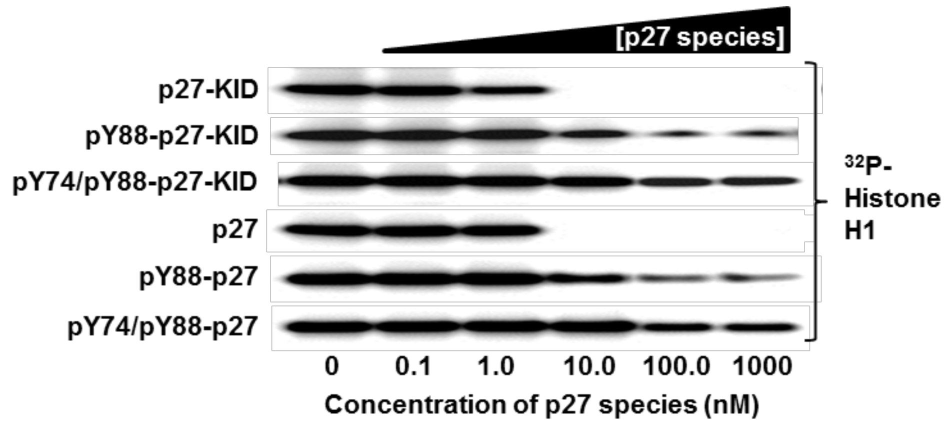

B

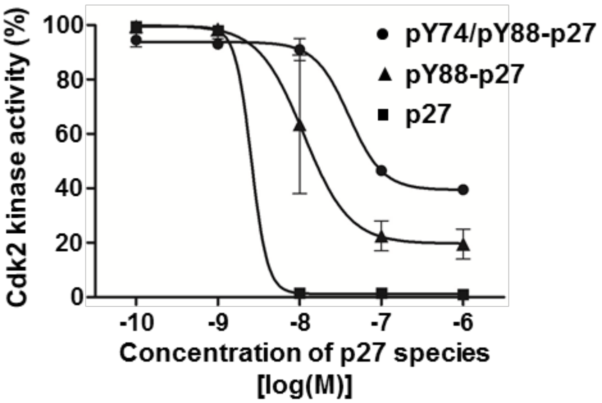

Supplementary Figure 1. Incremental phosphorylation of tyrosine residues in p27 exerts rheostat-like control over Cdk2/cyclin A activity.

(A) Representative results of kinase activity assays for Cdk2/cyclin A in the presence of increasing concentrations of unphosphorylated and mono and dual Y phosphorylated p27-KID and p27. Autoradiography was used to monitor incorporation of ^{32}P-labeled phosphate into the substrate, Histone H1, which was resolved using SDS-PAGE. The regions of the autoradiograms corresponding to the Histone H1 protein in the gels are shown. The experiments were performed in triplicate. The results for p27-KID, pY88-p27-KID and pY74/pY88-p27-KID are quantified in Fig. 2A as average kinase activity (± standard deviation of the mean, n=3) relative to that in the absence of p27-KID. (B) Quantification of the results presented in (A) for mono and dual Y phosphorylated p27. The results are quantified as in Fig. 2A.

A

B

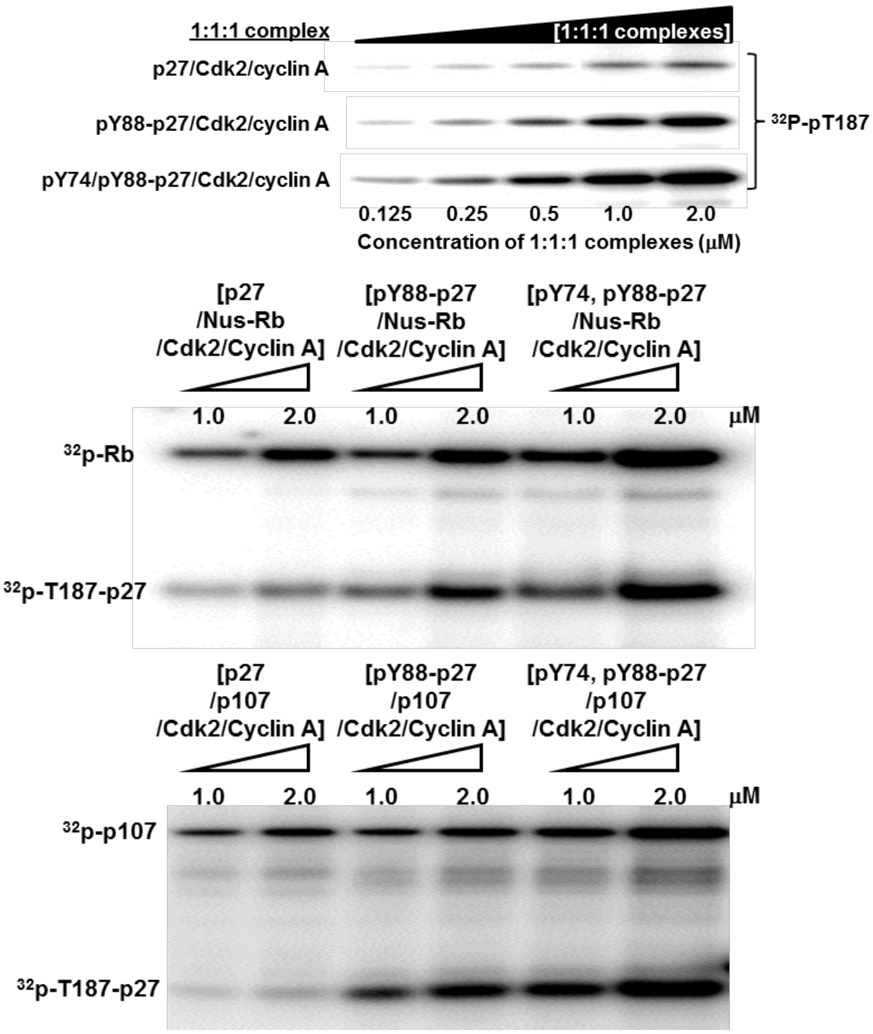

Supplementary Figure 2. Incremental phosphorylation of tyrosine residues in p27 exerts rheostat-like control over Cdk2/cyclin A and promotes phosphorylation of p27 on T187.

(A) Representative results of kinase activity assays for different concentrations of 1:1:1 complexes of Cdk2/cyclin A with p27, pY88-p27 or pY74/pY88-p27 with T187 (of p27) as the substrate. Autoradiography was used to monitor incorporation of ^{32}P-labeled phosphate into T187 of p27, which was resolved using SDS-PAGE. The regions of the autoradiograms corresponding to the p27 protein in the gels are shown. The experiments were performed in triplicate. These results are quantified in Fig. 2C as average kinase activity (± standard deviation of the mean, n=3) relative to that for the highest concentration of pY74/pY88-p27/Cdk2/cyclin A. (B) Kinase activity assays for 1.0 and 2.0 μM 1:1:1 complexes of Cdk2/cyclin A with p27, pY88-p27 or pY74/pY88-p27 with equimolar amounts of either Nus-tagged Rb C-terminus (Nus-Rb) or GST/His-tagged p107 C-terminus (p107) as “in trans” Cdk2 substrates. Autoradiography was used to monitor incorporation of ^{32}P-labeled phosphate into T187 of p27 and Nus-Rb or p107, which were resolved using SDS-PAGE. The regions of the autoradiograms corresponding to the p27 and Nus-Rb or p107 proteins in the gels are shown. The results shown in Figure 2D depict the average fold stimulation of incorporation of ^{32}P into each substrate as a function of the phosphorylation statues of p27. Error bars depict the standard deviation for the replicates at 1.0 and 2.0 μM 1:1:1 complexes (p27/Cdk2/cyclin A) with Nus-Rb or p107 as the inter-molecular substrate. In each incubation the concentration of the intermolecular substrate (Nus-Rb or p107) matches that of the p27/Cdk2/cyclin A complex.

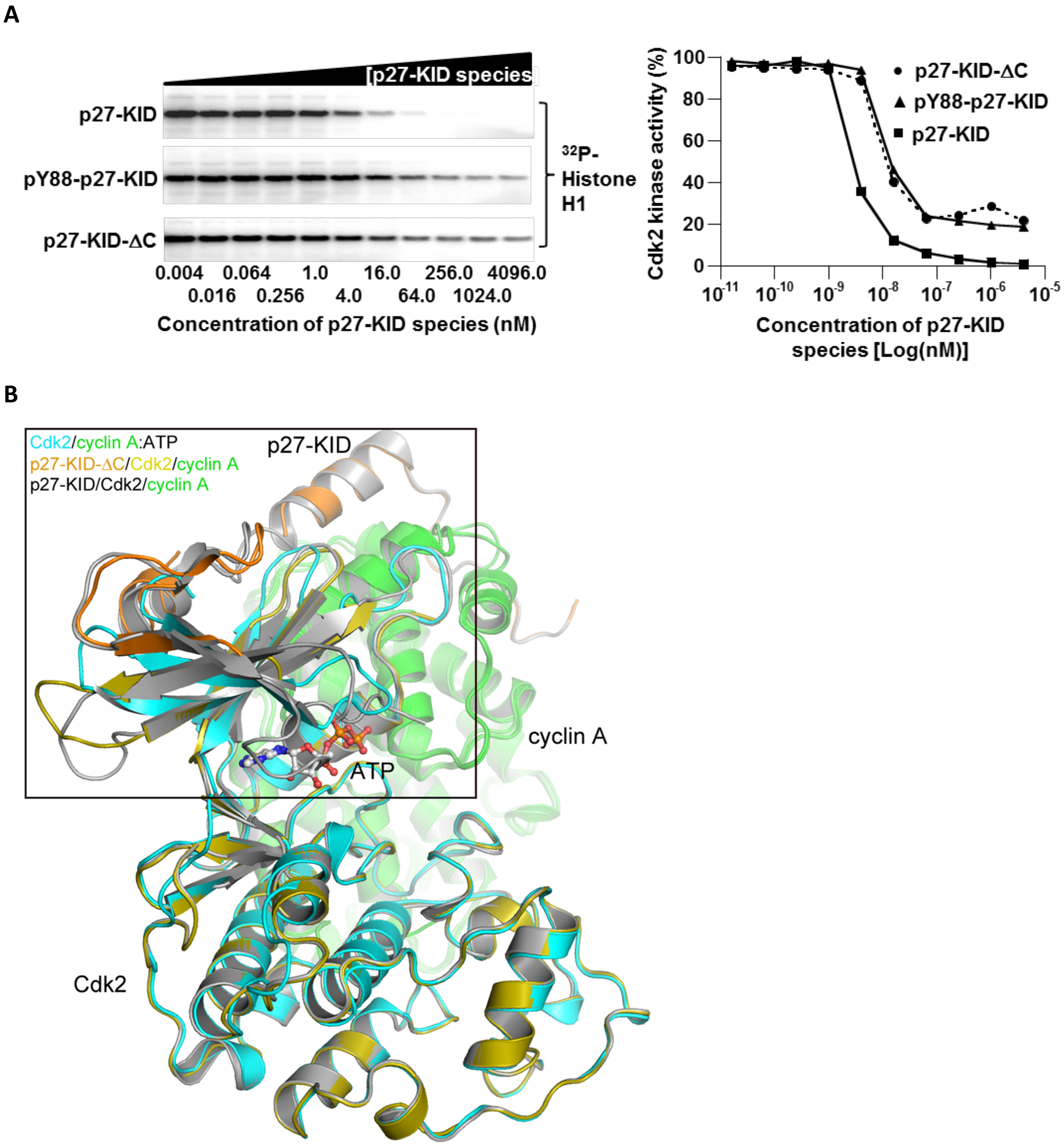

Supplementary Figure 3. Ejection of pY88 and the residue 83-89 region from the Cdk2 active site is mimicked by truncation of p27-KID at residue 79. (A) Left, representative results of kinase activity assays for Cdk2/cyclin A in the presence of increasing concentrations of p27-KID, pY88-p27-KID or p27-KID-ΔC (p27-KID with residues 80-94 deleted). Autoradiography was used to monitor incorporation of ^{32}P-labeled phosphate into the substrate, Histone H1, which was resolved using SDS-PAGE. The regions of the autoradiograms corresponding to the Histone H1 protein in the gels are shown. The experiments were performed in triplicate. Right, quantification of the results on the left showing average kinase activity (± standard deviation of the mean, n=3) relative to that for 4 pM p27-KID. (B) Superposition of the structures of Cdk2/cyclin A bound to ATP (PDB:1JST; Cdk2/cyclin A:ATP), p27-KID bound to Cdk2/cyclin A (PDB:1JSU; p27-KID/Cdk2/cyclin A) and p27-KID-ΔC bound to Cdk2/cyclin A (p27-KID- ΔC/Cdk2/cyclin A, determined in this study). The structures are superimposed on backbone heavy atoms of cyclin A. The color code is indicated in the illustration and the boxed region is illustrated in Fig. 3B. The PyMOL Molecular Graphics System (Schrödinger, LLC) was used to prepare the illustration.

Supplementary Figure 4 (below). Multidimensional smFA histograms of p27/Cdk2/cyclin A at various phosphorylation states.

Two-dimensional histogram of scatter anisotropy (r_D) vs. $\langle\tau_{D(A)}\rangle_f$ for Bodipy labeled single Cys p27 variants in complex with Cdk2/Cyclin A. "Burstwise" analysis of A) C29, B) C40, C) C54, D) C75 and E) C93 variants. For all cases one dimensional projections for $\langle\tau_{D(A)}\rangle_f$ and anisotropy are also shown. Pure donor fluorescence (F_D) is corrected for background ($\langle B_G\rangle$ = 1.07 kHz,). Perrin's equation for r_{low} (blue) and r_{high} (purple) using $\tau_D^{(\mathrm{High\,r})}$ and $\tau_D^{(\mathrm{Low\,r})}$ are shown (Supplementary Table 2 and Supplementary Table 4). Rotational correlation times $\rho^{(\mathrm{Low\ rD})}$ and $\rho^{(\mathrm{High\ rD})}$ are given as numbers in purple and blue in the respective plot for the corresponding populations. Number of Bursts per dimension are shown on the top right corner for each sample.

A Cdk2/cyclin A/p27-C29

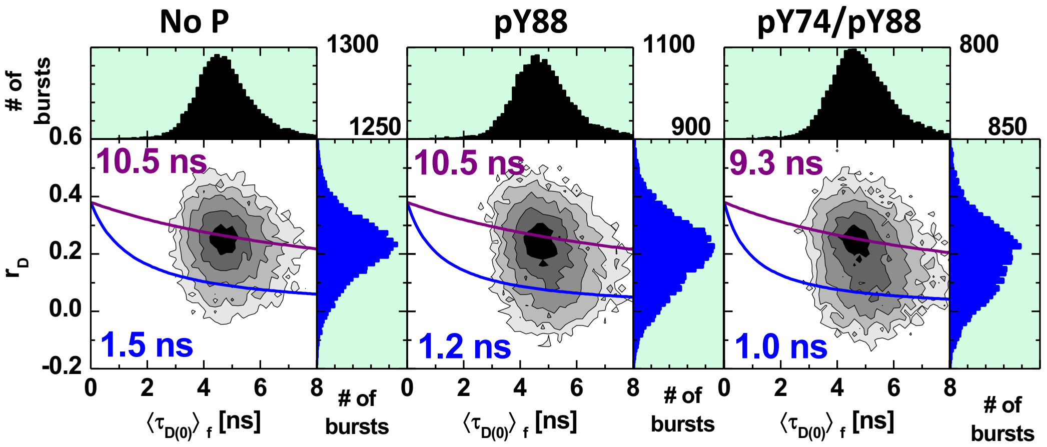

B Cdk2/cyclin A/p27-C40

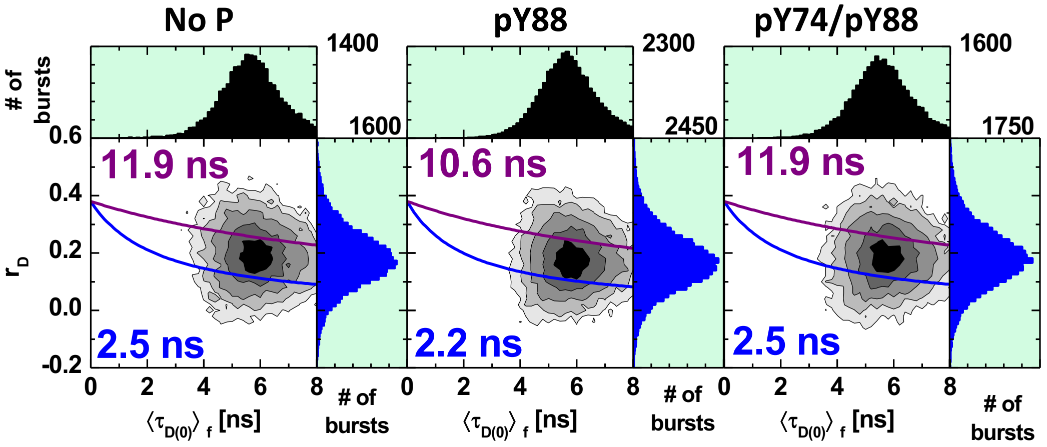

C **Cdk2/cyclin A/p27-C54**

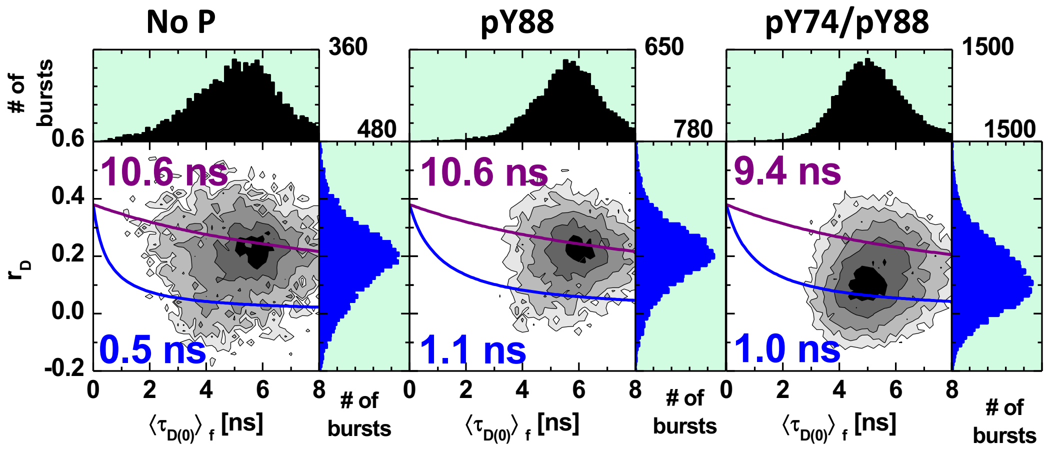

D **Cdk2/cyclin A/p27-C75**

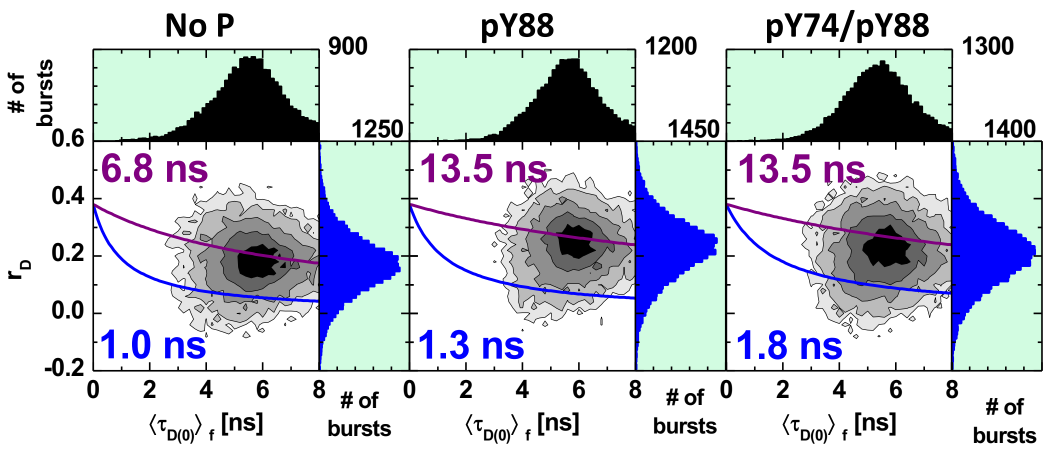

E **Cdk2/cyclin A/p27-C93**

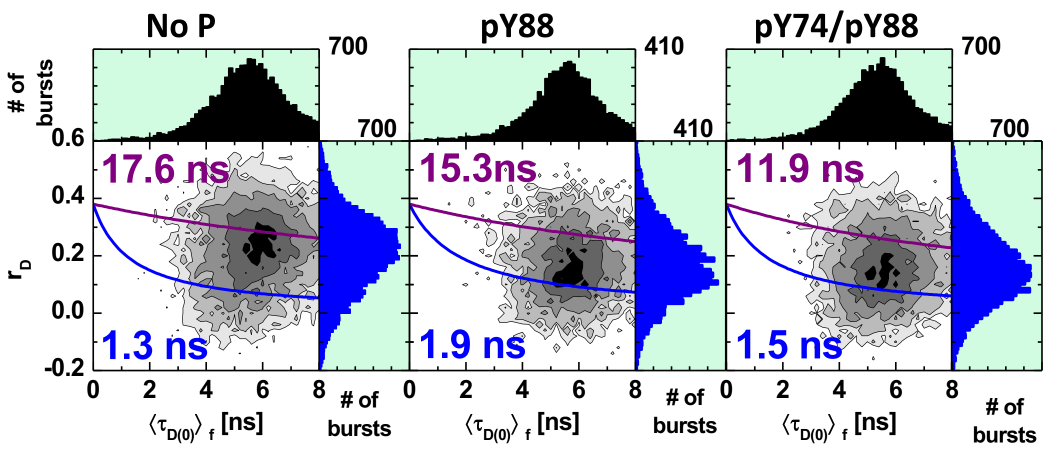

Supplementary Figure 5 (below). Multidimensional smFRET histograms of p27/Cdk2/cyclin A at various phosphorylated states.

Two-dimensional histogram F_D/F_A vs. lifetime of donor in the presence of acceptor $\langle\tau_{D(A)}\rangle_f$, and scatter corrected donor anisotropy (r_D) vs. $\langle\tau_{D(A)}\rangle_f$ for Cdk2/cyclin A/p27 with donor and acceptor dyes at various positions in "burstwise" mode. A) C29-54, B) C54-93 and C) C75-110. For all cases one dimensional projections for F_D/F_A, $\langle\tau_{D(A)}\rangle_f$ and anisotropy are also shown. Pure donor and acceptor fluorescence (F_D and F_A) are corrected for background ($\langle B_G\rangle$ = 1.57 kHz A, B) or 0.65 kHz for C), $\langle B_R\rangle$ = 0.94 kHz A,B) or 0.42 kHz C), spectral cross-talk (α = 1.7 %) and detection efficiency ratio (g_G/g_R = 0.8). Static FRET lines [Eq. (3)] are shown in blue. Dynamic FRET lines [Eq. (7)] between the Low and High $\langle R_{DA}\rangle_E$ states (Supplementary Tables 5-7) are shown in magenta. Light and dark horizontal lines mark the F_D/F_A ratio corresponding to the Low and High $\langle R_{DA}\rangle_E$ states. Perrin's equation with rotational correlation time ρ indicated in the 2D plot is shown as blue line.

A Cdk2/cyclin A/p27-C29-54

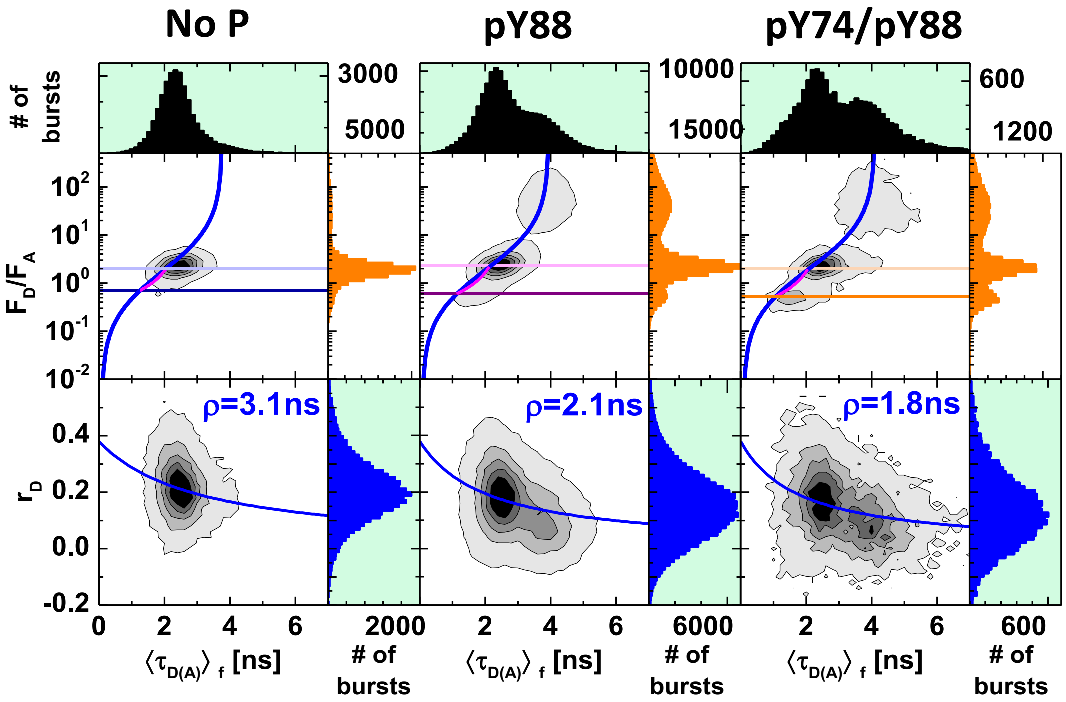

B Cdk2/cyclin A/p27-C54-93

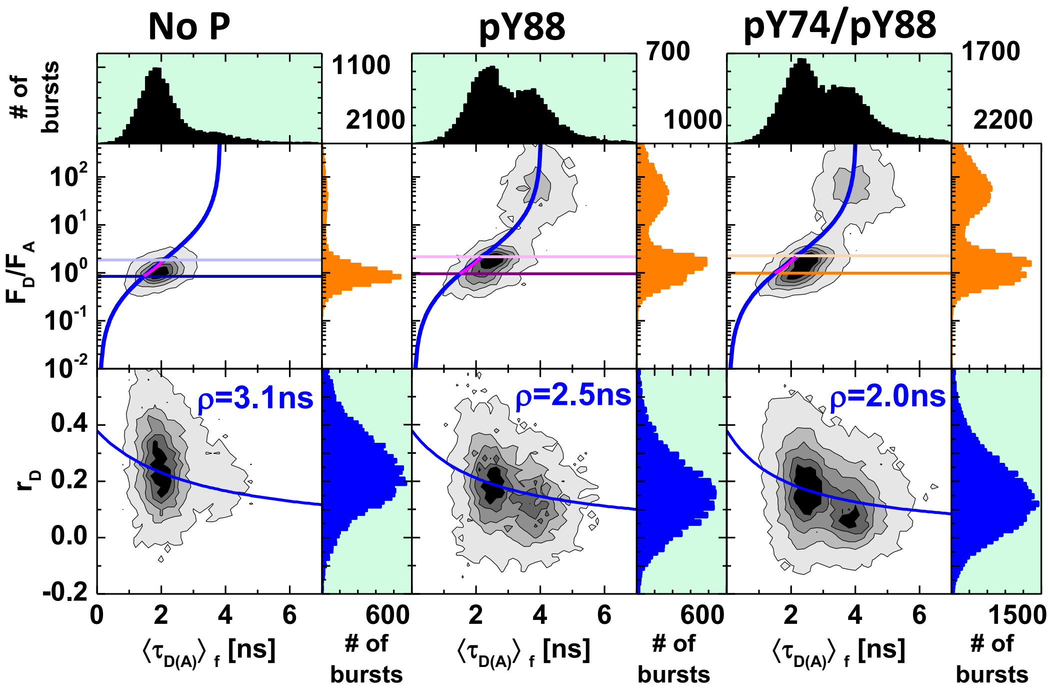

C Cdk2/cyclin A/p27-C75-110

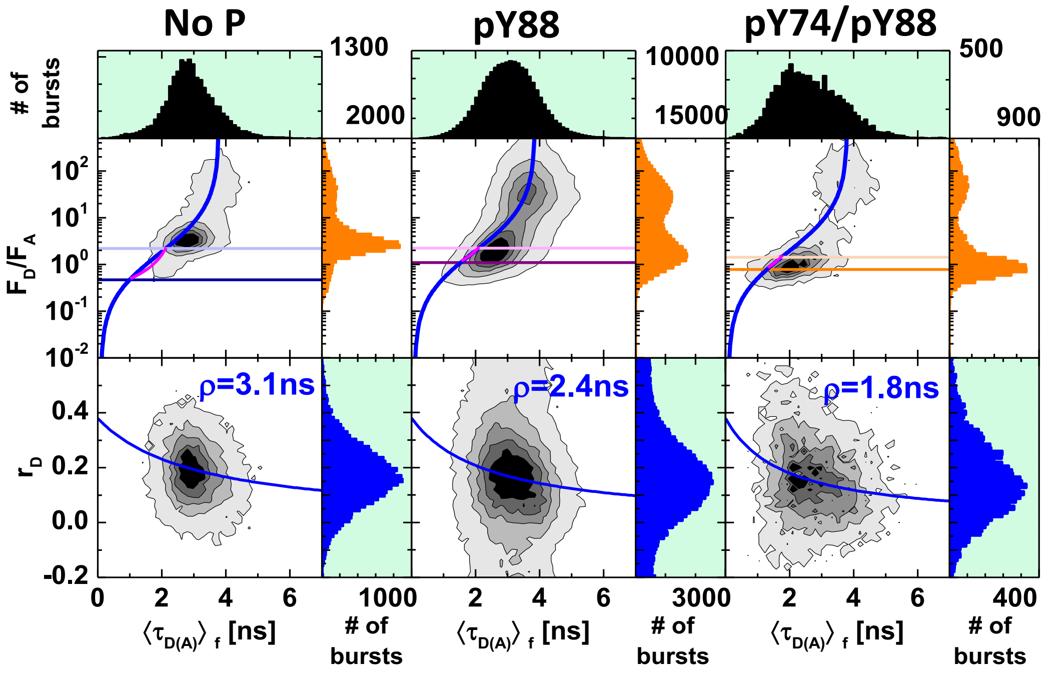

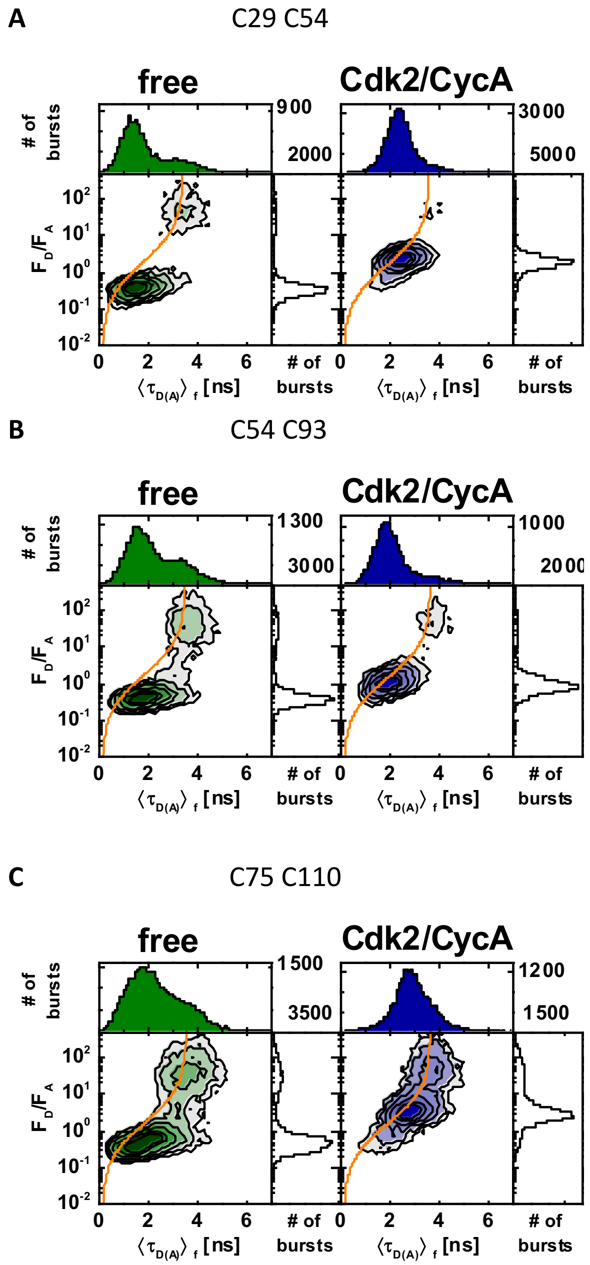

Supplementary Figure 6. smMFD of free vs. complex p27 with Cdk2/cyclin A.
Two-dimensional histogram F_D/F_A vs. lifetime of donor in the presence of acceptor $\langle\tau_{D(A)}\rangle_f$ for free and Cdk2/cyclin A/p27 complex with donor and acceptor dyes at various positions. **A)** C29-54, **B)** C54-93 and **C)** C75-110. For all cases one dimensional projections for F_D/F_a and $\langle\tau_{D(A)}\rangle_f$ are also shown. Pure donor and acceptor fluorescence (F_D and F_A) are corrected for background ($\langle B_G\rangle$ = 1.62 kHz (free) or 1.57 kHz (bound) **A,B)** or 0.65 kHz for **C)** $\langle B_R\rangle$ = 1.03 kHz (free) or 0.94 kHz (bound) **A,B)** or 0.42 kHz **C)**, spectral cross-talk (α = 1.7 %) and detection efficiency ratio (g_G/g_R = 0.8). Static FRET lines are shown in orange.

Supplementary Figure 7 (below). Filtered FCS Species auto and cross-correlation (sACF and sCCF) function of smFRET experiments for Cdk2/cyclin A/p27 samples.
Filtered Fluorescence Auto and Cross-Correlation, left sACF and right sCCF, respectively. Filters were selected by "burstwise" selection based on FD/FA arbitrary cutoffs to select low-FRET (LF) or high-FRET (HF) populations. The integrated fluorescence of these burst corresponds to two independent species. The two sCCF (right) (HF to LF and LF to HF) and the sACF where globally fit using Eq. (18) to determine the number of relaxation times and their amplitudes (Supplementary Table 9). Residuals of the fit for the sACF and sCCF are shown on top of each correlation curve. Similar treatment was used for the pY88, data not shown.

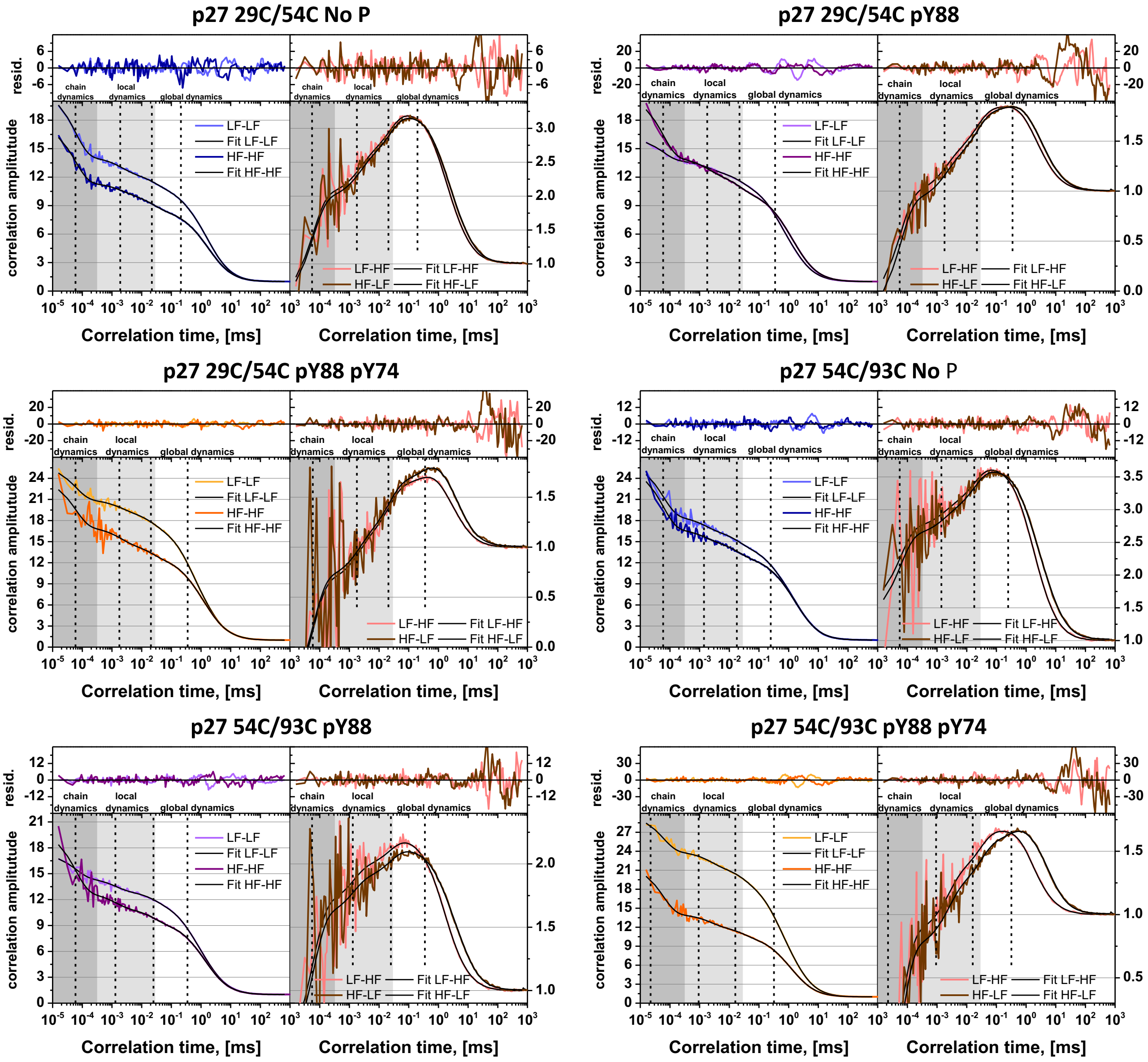

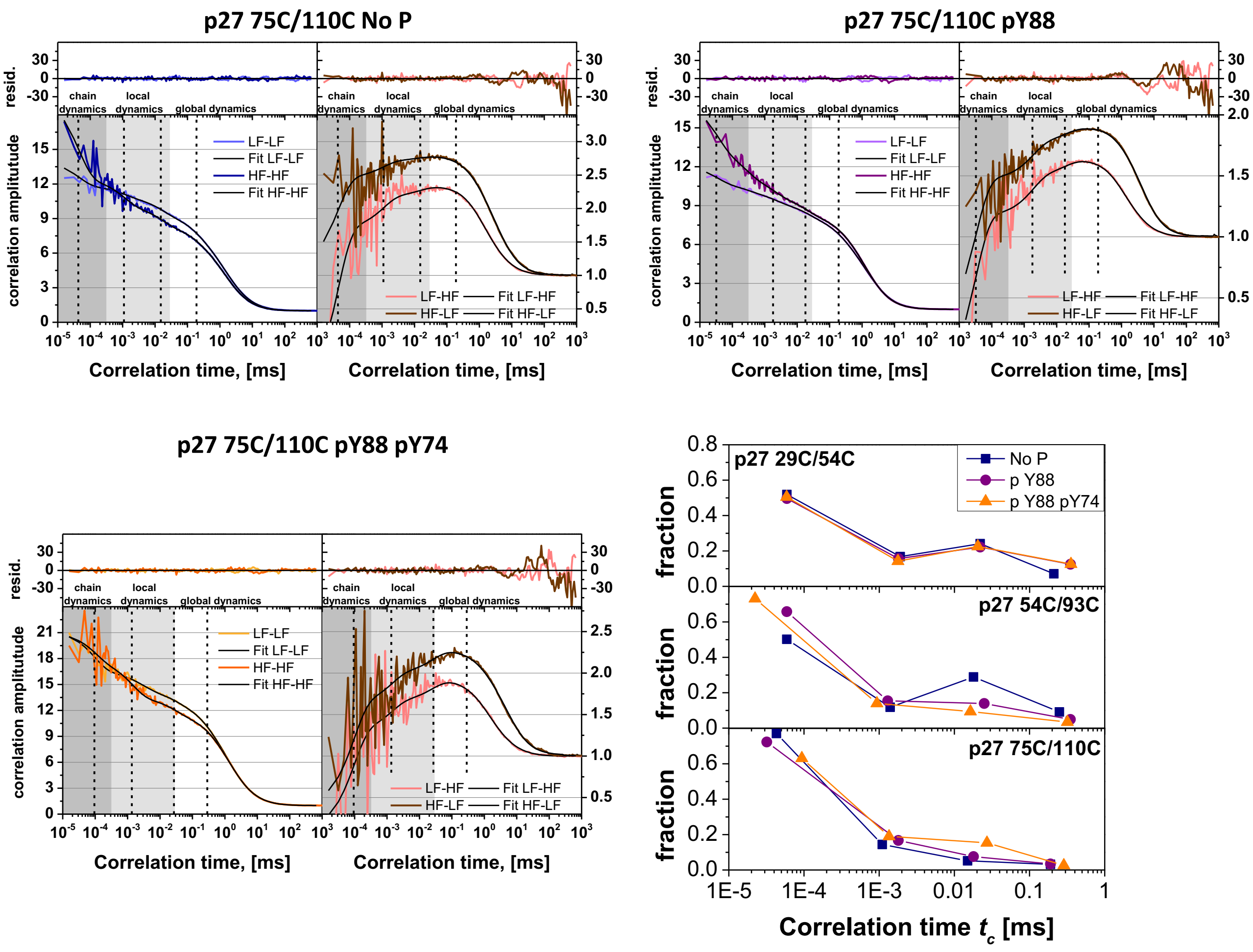
p27 75C/110C No P
p27 75C/110C pY88
p27 75C/110C pY88 pY74
resid.
correlation amplitude
chain dynamics
local dynamics
global dynamics
LF-LF
Fit LF-LF
HF-HF
Fit HF-HF
LF-HF
Fit LF-HF
HF-LF
Fit HF-LF
Correlation time, [ms]
p27 29C/54C
p27 54C/93C
p27 75C/110C
No P
p Y88
p Y88 pY74
fraction
Correlation time t_c [ms]

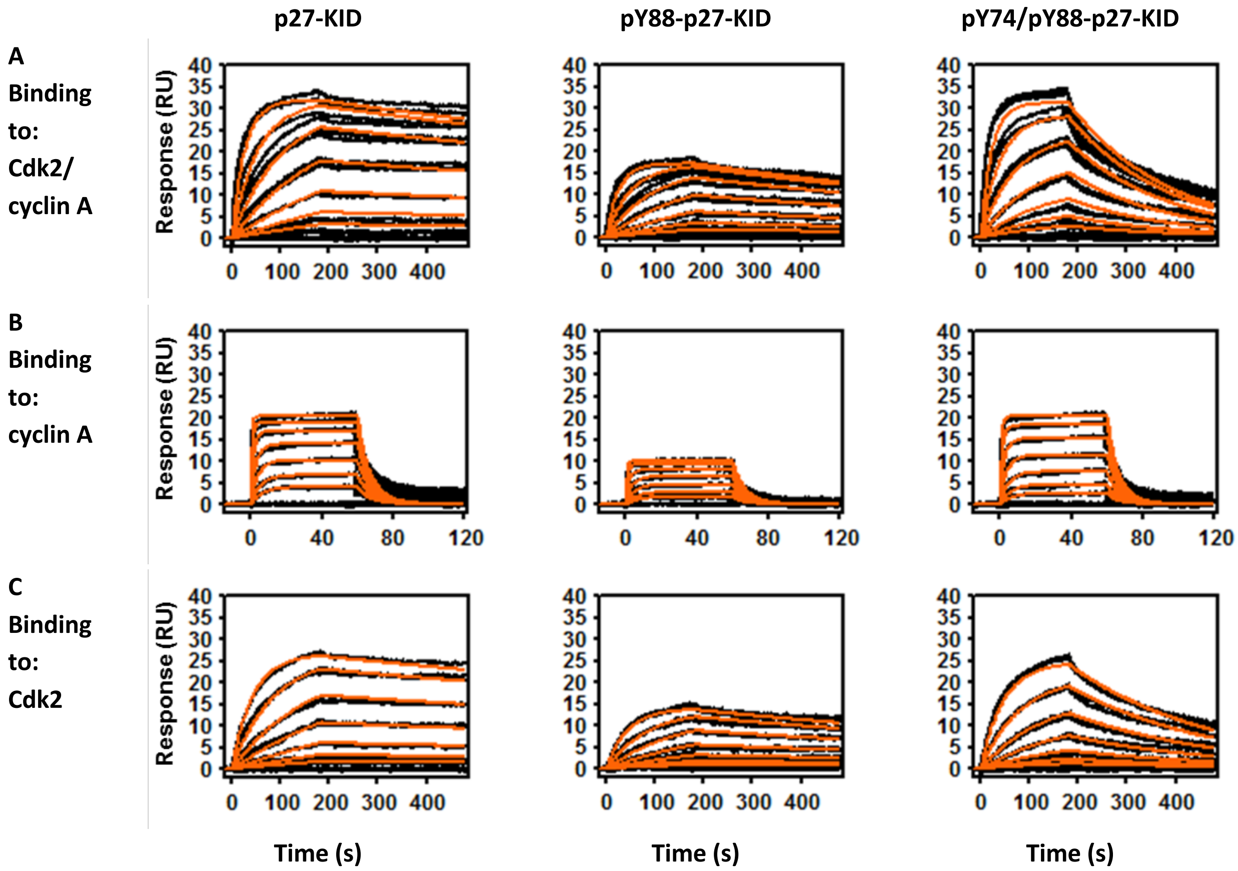

Supplementary Figure 8. Representative results for varied concentrations of Cdk2/cyclin A, cyclin A, or Cdk2 binding to p27-KID, pY88-p27-KID, or pY74/Y88-p27-KID separately immobilized on the sensor surface.

The results of triplicate injections are shown with fits of a 1:1 Langmuir interaction model shown as solid orange curves. The kinetic constants and K_D values derived from these analyses are provided in Supplementary Table 10. Sensograms showing the binding of p27-KID phosphoforms to the Cdk2/cyclin A complex, cyclin A individually, and Cdk2 individually are shown in panels A, B, and C, respectively.

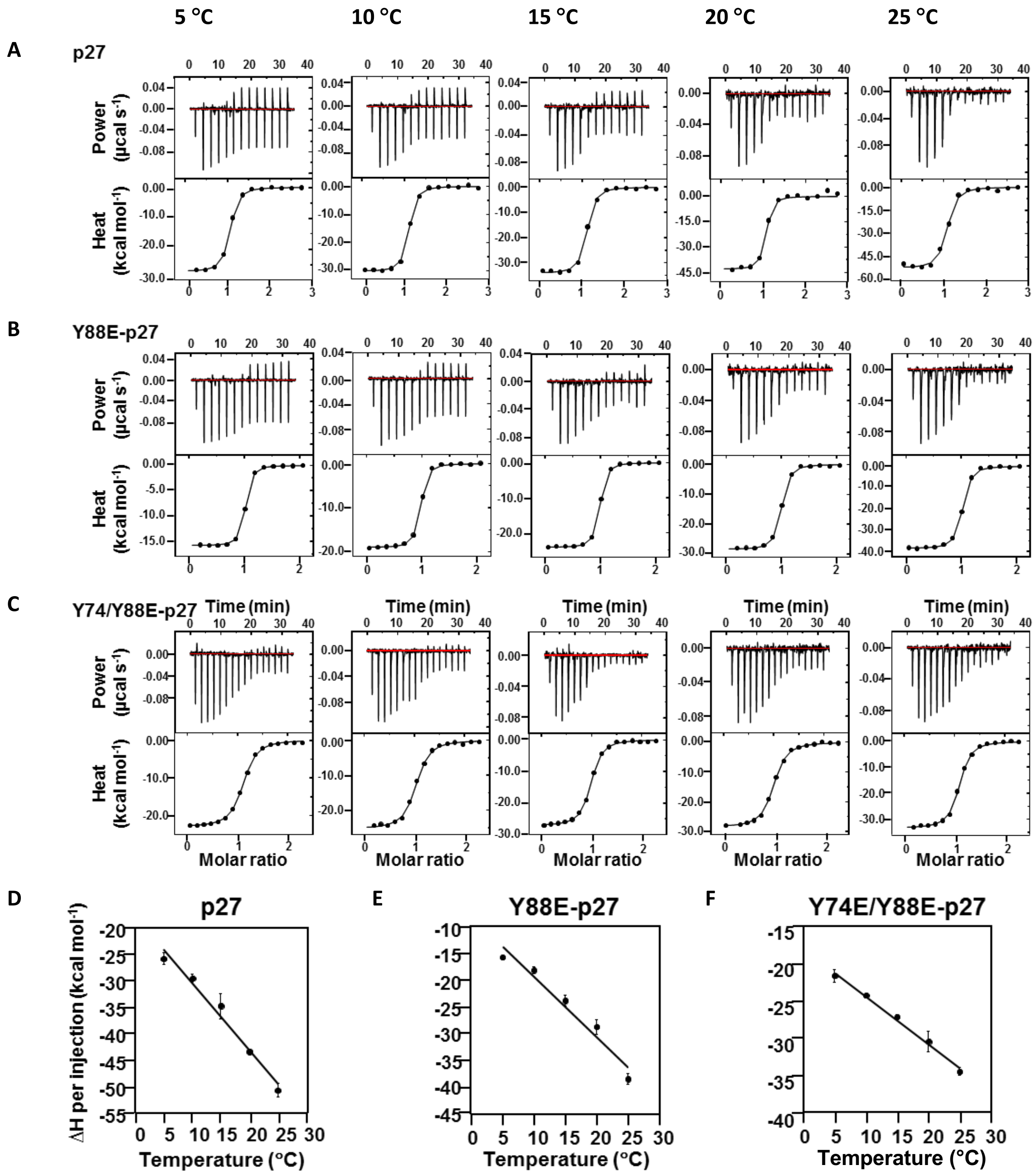

Supplementary Figure 9. Representative binding isotherms for injected p27 (A), Y88E-p27 (B), or Y74E/Y88E-p27 (C) binding to Cdk2/cyclin A at temperatures from 5 °C to 25 °C recorded using isothermal titration calorimetry (ITC). For each experiment, the upper panel shows the power and the lower panel the heat associated with each injection. The enthalpy of binding (ΔH) values derived from analysis of triplicate measurements using a 1:1 binding model are provided in Table 1. Error bars represent the standard deviation from the mean, n=3. Heat capacity change for binding (ΔC_p) values were determined from the slope of ΔH *versus* temperature plots (panels D, E, and F); ΔC_p values are provided in Table 1.

Supplementary Figure 10 (below). Multidimensional smFRET histograms of p27/Cdk2/Cyclin A with phosphomimetic variants.

Two-dimensional histogram F_D/F_A vs. lifetime of donor in the presence of acceptor $\langle\tau_{D(A)}\rangle_f$, and scatter corrected donor anisotropy (r_D) vs. $\langle\tau_{D(A)}\rangle_f$ for Cdk2/Cyclin A/p27 with donor and acceptor dyes at various positions with phosphomimetic mutations at positions E88 and E74/88. "Burstwise" mode of A) C29-54, B) C54-93 and C) C75-110 samples. For all cases one dimensional projections for F_D/F_A, $\langle\tau_{D(A)}\rangle_f$ and anisotropy are also shown. Pure donor and acceptor fluorescence (F_D and F_A) are corrected for background ($\langle B_G\rangle$ = 1.80 kHz, $\langle B_R\rangle$ = 0.27 kHz), spectral cross-talk (α = 1.7%), direct acceptor excitation (β = 1.3 %) and detection efficiency ratio (g_G/g_R = 0.8). Values for "No P" samples are given in the legend to Supplementary Figure 5. Static FRET lines (Eq. (3)) are shown in blue. Dynamic FRET lines (Eq. (7)) between the Low and High $\langle R_{DA}\rangle_E$ states (Table 5,7) are shown in magenta. Perrin's equation with rotational correlation time ρ indicated in the 2D plot is shown as blue line.

A Cdk2/cyclin A/p27-C29-54

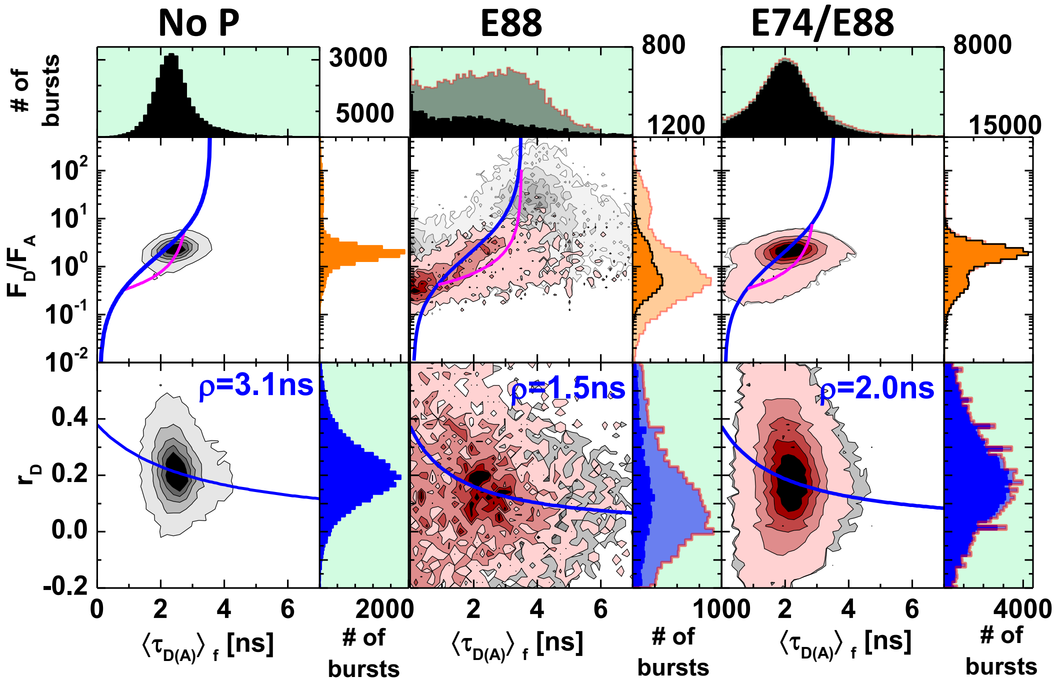

B Cdk2/cyclin A/p27-C54-93

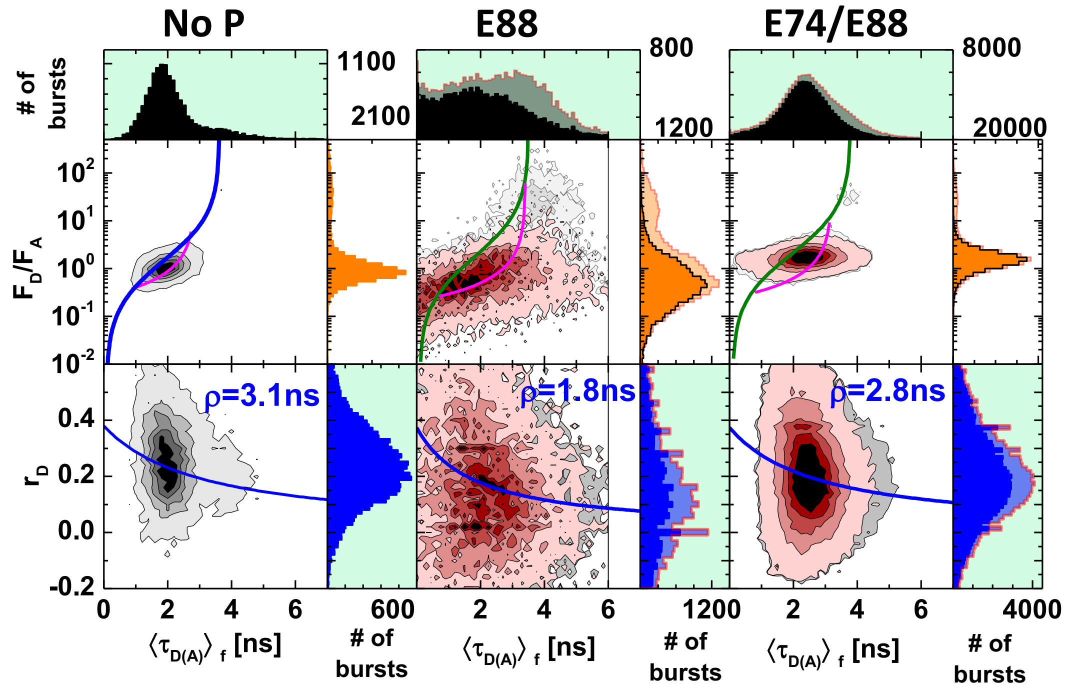

C Cdk2/cyclin A/p27-C75-110

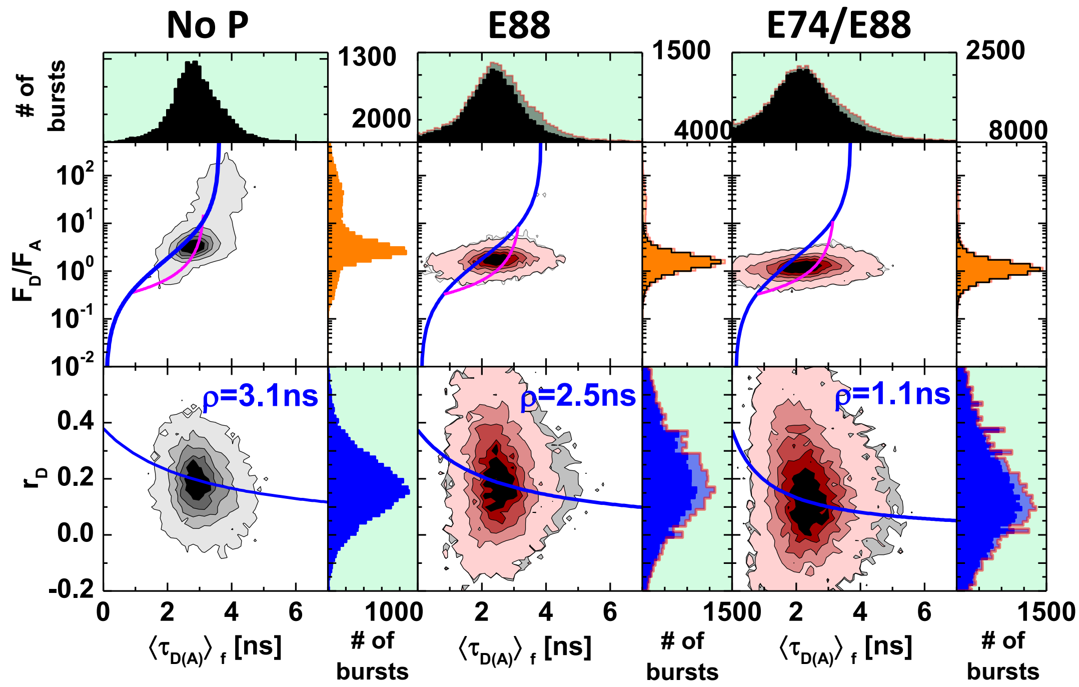

A

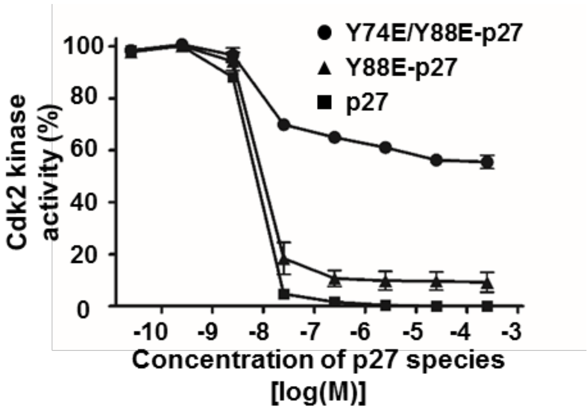

B

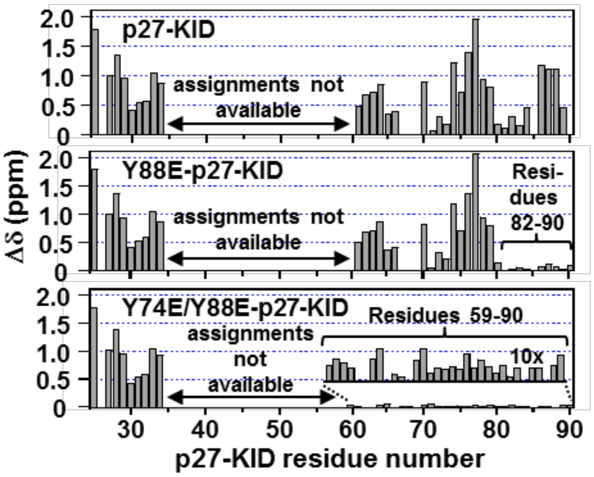

Supplementary Figure 11. The effects of mono and dual tyrosine phosphorylation on regulation of Cdk2 by p27 can be mimicked by mutation of Y88, and Y74 and Y88, to glutamate (E).

(A) Results of kinase activity assays for Cdk2/cyclin A in the presence of increasing concentrations of p27, Y88E-p27 or Y74E/Y88E-p27. Autoradiography was used to quantify incorporation of ^{32}P-labeled phosphate into the substrate, Histone H1, which was resolved using SDS-PAGE (not shown). The experiments were performed in triplicate and the results quantified as average kinase activity (± standard deviation of the mean) relative to that in the presence of the lowest concentration of p27 expressed as percentage activity values. (B) NMR analysis of the influence of tyrosine to glutamate mutagenesis on interactions between p27-KID and Cdk2/cyclin A. Chemical shift differences for residues in unmutated (top) and tyrosine to glutamate mutated (Y88E-p27-KID, middle; and Y74E/Y88E-p27-KID, bottom) p27-KID bound to Cdk2/cyclin A. Residues near Y88 and within the entire D2 subdomain adopt free state-like conformations in Y88E-p27-KID and Y74E/Y88E-p27-KID, respectively. $\Delta\delta$ values were calculated using the equation: $\Delta\delta = [(\Delta\delta\ ^{1}H_{N})^2 + 0.0289 \times (\Delta\delta\ ^{15}N_{H})^2]^{1/2}$.

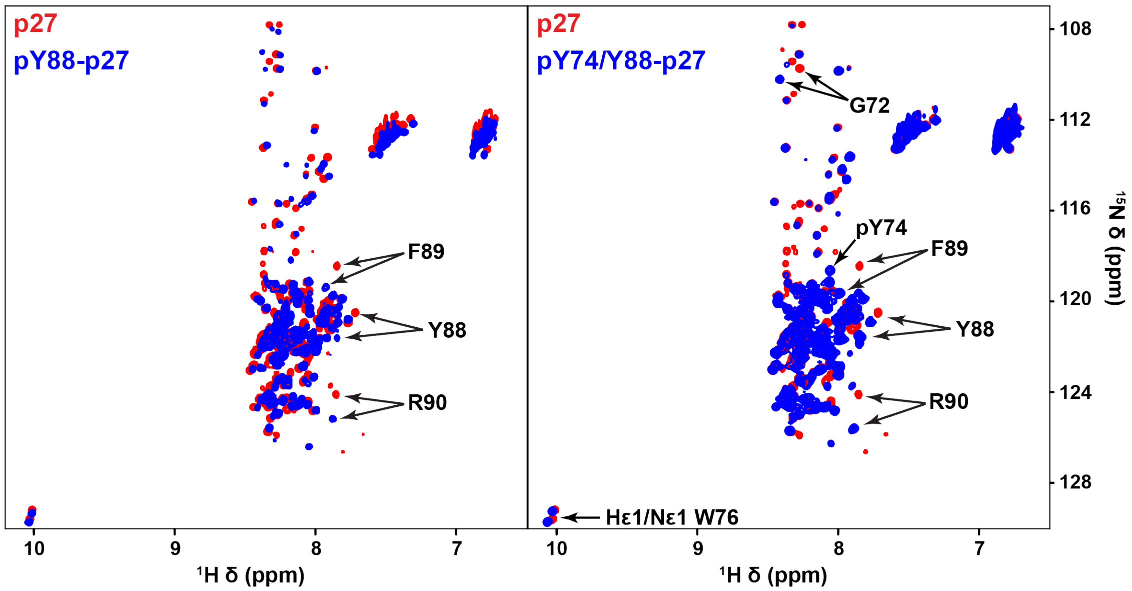

Supplementary Figure 12. Confirmation of the homogeneity of phosphorylated p27 for biochemical experiments.

^{1}H-^{15}N HSQC spectra of purified and homogenous preparations of phosphorylated forms of p27 used in biochemical experiments. In each case the unmodified reference spectrum is shown in red with the various phosphorylated forms depicted in blue. Signature resonance shifts used to follow phosphorylation are indicated.

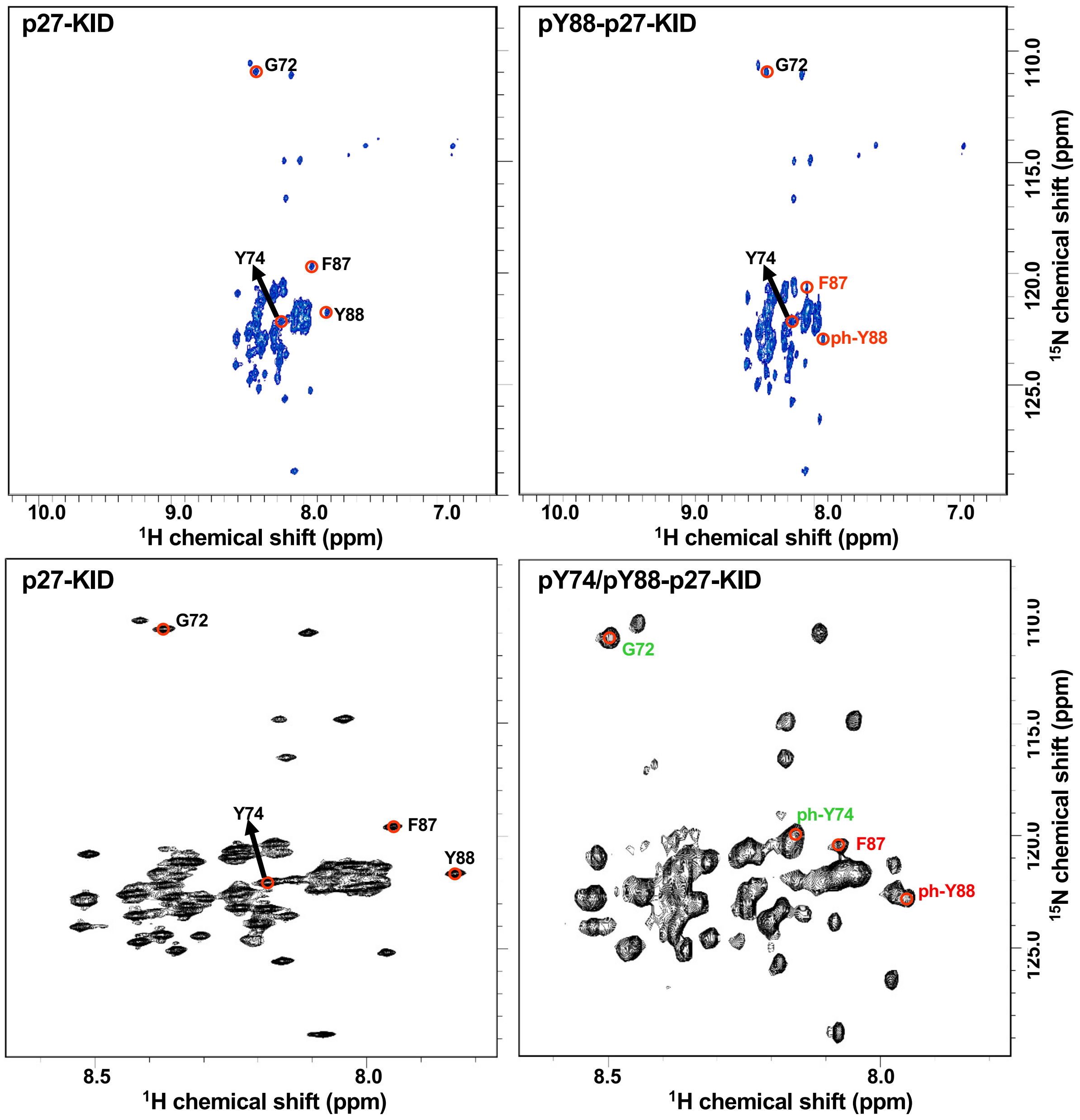

Supplementary Figure 13. Confirmation of the homogeneity of phosphorylated p27-KID for biochemical, SPR, and NMR experiments

^{1}H-^{15}N HSQC spectra of purified and homogenous preparations of phosphorylated forms of p27 used in biochemical, SPR and NMR experiments. Signature resonance shifts used to follow phosphorylation are indicated.

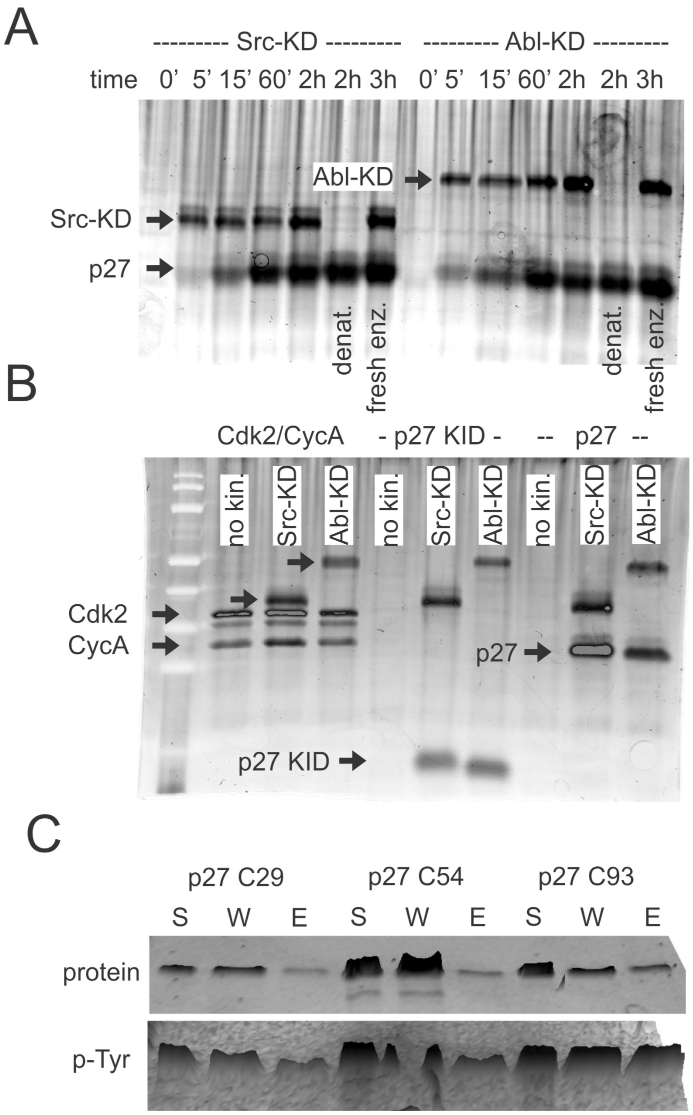

Supplementary Figure 14. Stoichiometric phosphorylation of p27 by Abl and Src kinases

Kinetics and completeness of phosphorylation is shown by several lines of evidence. (A) Both Src-KD and Abl-KD, under the given conditions, completely phosphorylate full-length p27. p27 was incubated with either Src-KD or Abl-KD for the time indicated, run on an SDS-PAGE and Western-blotted by a Phospho-Tyrosine Mouse mAb (Bioke). Both reactions reached saturation in about 60 min, and removal of kinase by heat denaturation (denat., at 2h) and the addition of a new batch of enzyme (fresh enz.) did not lead to further phosphorylation. (B) Effective phosphorylation of p27and p27-KID by Src-KD and Abl-KD. Phosphorylation of Cdk2/CycA, p27-KID and full length p27 was carried out as above by both Src-KD and Abl-KD, and SDS gels (with sample "no kin." For controls run without added kinase) were Western blotted by the above anti-phospho-Tyr antibody. Apparently, neither Src-KD, nor Abl-KD phosphorylated Cdk2 and CycA, but completely phosphorylated both p27-KID and full-length p27. Complete (stoichiometric) phosphorylation by Src-KD is seen by the complete mobility shift of p27-KID (and, although less apparent, also of p27). As shown next, this is then proven by purification on a phospho-specific membrane. (C) For three fluorescence labelled variants of p27 (p27-C29, -C54 and -C93), we show further enrichment for phosphorylated protein following phosphorylation, by binding the sample (S) to the membrane of a Pro-Q® Diamond Phosphoprotein Enrichment Kit (Invitrogen), extensive washing (W) and specific elution (E) of phosphorylated protein. We applied conditions under which most protein (about 90%) eluted in the washing step (see upper gel picture, stained for protein), ensuring a further very strong enrichment of finally eluted protein for phosphorylated molecules (cf. lower picture of Western blot by a p-Tyr specific antibody). From comparing intensities, at least 98% of the protein eluted from the membrane is phosphorylated.

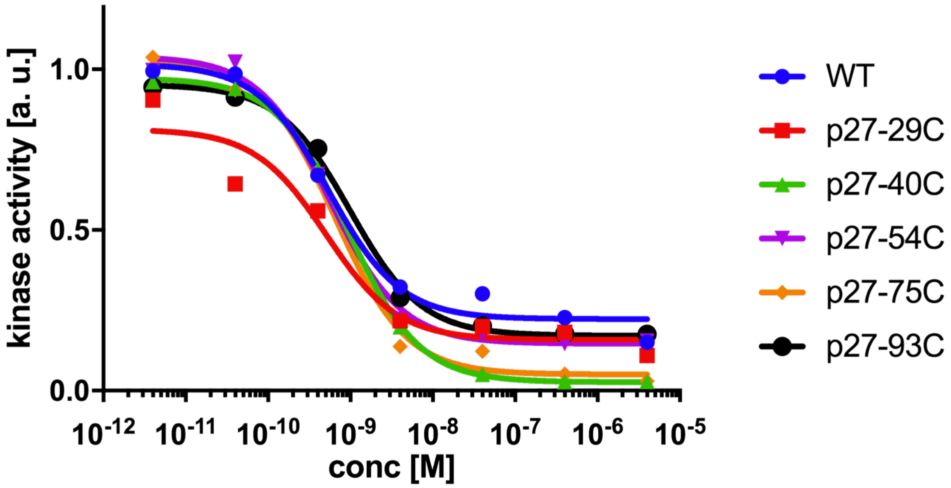

Supplementary Figure 15. p27 cysteine mutants inhibit Cdk2/cyclin similarly to WT.
Quantification of kinase assays demonstrating that the p27 single cysteine mutants inhibit Cdk2/cyclin A to a similar degree as WT. Autoradiography was used to monitor incorporation of ^{32}P-labeled phosphate into the substrate, Histone H1, which was resolved using SDS-PAGE. Data were normalized to the extent of substrate phosphorylation in the absence of p27.

Supplementary References

1. Maus M*, et al.* An experimental comparison of the maximum likelihood estimation and nonlinear least-squares fluorescence lifetime analysis of single molecules. *Anal Chem* **73**, 2078-2086 (2001).

2. Sindbert S*, et al.* Accurate Distance Determination of Nucleic Acids via Forster Resonance Energy Transfer: Implications of Dye Linker Length and Rigidity. *Journal of the American Chemical Society* **133**, 2463-2480 (2011).

3. Kalinin S, Sisamakis E, Magennis SW, Felekyan S, Seidel CA. On the origin of broadening of single-molecule FRET efficiency distributions beyond shot noise limits. *J Phys Chem B* **114**, 6197-6206 (2010).

4. Schaffer J, Volkmer A, Eggeling C, Subramaniam V, Striker G, Seidel CAM. Identification of single molecules in aqueous solution by time-resolved fluorescence anisotropy. *J Phys Chem A* **103**, 331-336 (1999).

5. Koshioka M, Sasaki K, Masuhara H. Time-Dependent Fluorescence Depolarization Analysis in 3-Dimensional Microspectroscopy. *Appl Spectrosc* **49**, 224-228 (1995).

6. Antonik M, Felekyan S, Gaiduk A, Seidel CA. Separating structural heterogeneities from stochastic variations in fluorescence resonance energy transfer distributions via photon distribution analysis. *J Phys Chem B* **110**, 6970-6978 (2006).

7. Kalinin S, Felekyan S, Antonik M, Seidel CAM. Probability distribution analysis of single-molecule fluorescence anisotropy and resonance energy transfer. *Journal of Physical Chemistry B* **111**, 10253-10262 (2007).

8. Kalinin S, Felekyan S, Valeri A, Seidel CA. Characterizing multiple molecular States in single-molecule multiparameter fluorescence detection by probability distribution analysis. *J Phys Chem B* **112**, 8361-8374 (2008).

9. Soong TT. *Fundamentals of Probability and Statistics for Engineers*. Wiley VCH (2004).

10. Felekyan S, Kalinin S, Sanabria H, Valeri A, Seidel CAM. Filtered FCS: species auto- and cross-correlation functions highlight binding and dynamics in biomolecules. *ChemPhysChem* **13**, 1036-1053 (2012).

11. Böhmer M, Wahl M, Rahn HJ, Erdmann R, Enderlein J. Time-resolved fluorescence correlation spectroscopy. *Chem Phys Lett* **353**, 439-445 (2002).

12. Elson EL, Magde D. Fluorescence Correlation Spectroscopy. I. Conceptual Basis and Theory. *Biopolymers* **13**, 1-27 (1974).

13. Felekyan S, Sanabria H, Kalinin S, Kühnemuth R, Seidel CAM. Analyzing Förster resonance energy transfer (FRET) with fluctuation algorithms. *Methods Enzymol* **519**, 39-85 (2013).

14. Felekyan S, Kalinin S, Sanabria H, Valeri A, Seidel CA. Filtered FCS: species auto- and cross-correlation functions highlight binding and dynamics in biomolecules. *Chemphyschem* **13**, 1036-1053 (2012).

15. Sindbert S*, et al.* Accurate distance determination of nucleic acids via Forster resonance energy transfer: implications of dye linker length and rigidity. *J Am Chem Soc* **133**, 2463-2480 (2011).

16. Grimmler M*, et al.* Cdk-inhibitory activity and stability of p27(Kip1) are directly regulated by oncogenic tyrosine kinases. *Cell* **128**, 269-280 (2007).
