# Supplementary material for: Dynamic anticipation by Cdk2/Cyclin A-bound p27 mediates signal integration in cell cycle regulation
Source: Nat Commun. 2019 Apr 11;10:1676. doi: 10.1038/s41467-019-09446-w (PMC6459857; doi:10.1038/s41467-019-09446-w)
Supplement: Supplementary file 3 — Reporting Summary Checklist [file 41467_2019_9446_MOESM3_ESM.pdf]

nature research

Corresponding author(s): Richard Kriwacki

Last updated by author(s): Feb 18, 2019

Reporting Summary

Nature Research wishes to improve the reproducibility of the work that we publish. This form provides structure for consistency and transparency in reporting. For further information on Nature Research policies, see Authors & Referees and the Editorial Policy Checklist.

Statistics

For all statistical analyses, confirm that the following items are present in the figure legend, table legend, main text, or Methods section.

n/a	Confirmed	
☐	☒	The exact sample size (*n*) for each experimental group/condition, given as a discrete number and unit of measurement
☐	☒	A statement on whether measurements were taken from distinct samples or whether the same sample was measured repeatedly
☐	☒	The statistical test(s) used AND whether they are one- or two-sided<br>*Only common tests should be described solely by name; describe more complex techniques in the Methods section.*
☒	☐	A description of all covariates tested
☐	☒	A description of any assumptions or corrections, such as tests of normality and adjustment for multiple comparisons
☐	☒	A full description of the statistical parameters including central tendency (e.g. means) or other basic estimates (e.g. regression coefficient) AND variation (e.g. standard deviation) or associated estimates of uncertainty (e.g. confidence intervals)
☐	☒	For null hypothesis testing, the test statistic (e.g. *F*, *t*, *r*) with confidence intervals, effect sizes, degrees of freedom and *P* value noted<br>*Give P values as exact values whenever suitable.*
☒	☐	For Bayesian analysis, information on the choice of priors and Markov chain Monte Carlo settings
☒	☐	For hierarchical and complex designs, identification of the appropriate level for tests and full reporting of outcomes
☒	☐	Estimates of effect sizes (e.g. Cohen's *d*, Pearson's *r*), indicating how they were calculated

Our web collection on statistics for biologists contains articles on many of the points above.

Software and code

Policy information about availability of computer code

Data collection	All software used in data collection is described in the Online Methods section of the manuscript.
Data analysis	The software used to perform the smMFD analysis, written in house, can be downloaded from http://www.mpc.hhu.de/software.html.

For manuscripts utilizing custom algorithms or software that are central to the research but not yet described in published literature, software must be made available to editors/reviewers. We strongly encourage code deposition in a community repository (e.g. GitHub). See the Nature Research guidelines for submitting code & software for further information.

Data

Policy information about availability of data

All manuscripts must include a data availability statement. This statement should provide the following information, where applicable:

- Accession codes, unique identifiers, or web links for publicly available datasets
- A list of figures that have associated raw data
- A description of any restrictions on data availability

The atomic coordinates for the Cdk2/cyclin A/p27-KID-ΔC complex have been deposited and validated by the Protein Data Bank (PDB 6ATH) for release upon publication.

Field-specific reporting

Please select the one below that is the best fit for your research. If you are not sure, read the appropriate sections before making your selection.

☒ Life sciences ☐ Behavioural & social sciences ☐ Ecological, evolutionary & environmental sciences

October 2018

Life sciences study design

All studies must disclose on these points even when the disclosure is negative.

Sample size	Biochemical experiments were generally performed with 3 biological replicates. Exceptions to this are described in the methods.
Data exclusions	No data were excluded from the analysis.
Replication	Reproducibility was confirmed with independent experiments.
Randomization	Randomization of participants is not relevant to these biochemical and biophysical experiments.
Blinding	Samples were not blinded in these studies due to the small numbers of experimenters performing each experiment.

Reporting for specific materials, systems and methods

We require information from authors about some types of materials, experimental systems and methods used in many studies. Here, indicate whether each material, system or method listed is relevant to your study. If you are not sure if a list item applies to your research, read the appropriate section before selecting a response.

Materials & experimental systems

n/a	Involved in the study	
☐	☒	Antibodies
☒	☐	Eukaryotic cell lines
☒	☐	Palaeontology
☒	☐	Animals and other organisms
☒	☐	Human research participants
☒	☐	Clinical data

Methods

n/a	Involved in the study	
☒	☐	ChIP-seq
☒	☐	Flow cytometry
☒	☐	MRI-based neuroimaging

Antibodies

Antibodies used	Phospho-Tyrosine Mouse mAb (Bioke)
Validation	Phospho-Tyrosine Mouse mAb (P-Tyr-102) #9416. Phospho-Tyrosine Mouse mAb (P-Tyr-102) is a high affinity IgG1 monoclonal antibody. ELISAs using a wide variety of phospho-peptides show that P-Tyr-102 binds phospho-Tyr in a manner largely independent of the surrounding amino acid sequence.<br><br>2D gel western blot analysis of pervanadate-treated cell extracts also shows that P-Tyr-102 interacts with a broad range of tyrosine-phosphorylated proteins. P-Tyr-102's fine specificity in terms of the sequence context in which it can recognize phospho-tyrosine seems to differ slightly from that of P-Tyr-100 #9411. P-Tyr-102 does not recognize peptides containing phospho-Ser or phospho-Thr. (U.S. Patent No's.: 6,441,140; 6,982,318; 7,259,022; 7,344,714; U.S.S.N. 11,484,485; and all foreign equivalents.)
