# Supplementary material for: Dynamic anticipation by Cdk2/Cyclin A-bound p27 mediates signal integration in cell cycle regulation
Source: Nat Commun. 2019 Apr 11;10:1676. doi: 10.1038/s41467-019-09446-w (PMC6459857; doi:10.1038/s41467-019-09446-w)
Supplement: Supplementary file 4 — Source Data [file 41467_2019_9446_MOESM4_ESM.zip › 147848_2_related_ms_3589277_pnc75v (1).docx]

**Dynamic anticipation by Cdk2/Cyclin A-bound p27 mediates signal integration in cell cycle regulation**

M. Tsytlonok, H. Sanabria, Y. Wang *et al.*

**Source Data**

**Annotated complete phospho-images corresponding to Figure 2A.**

**
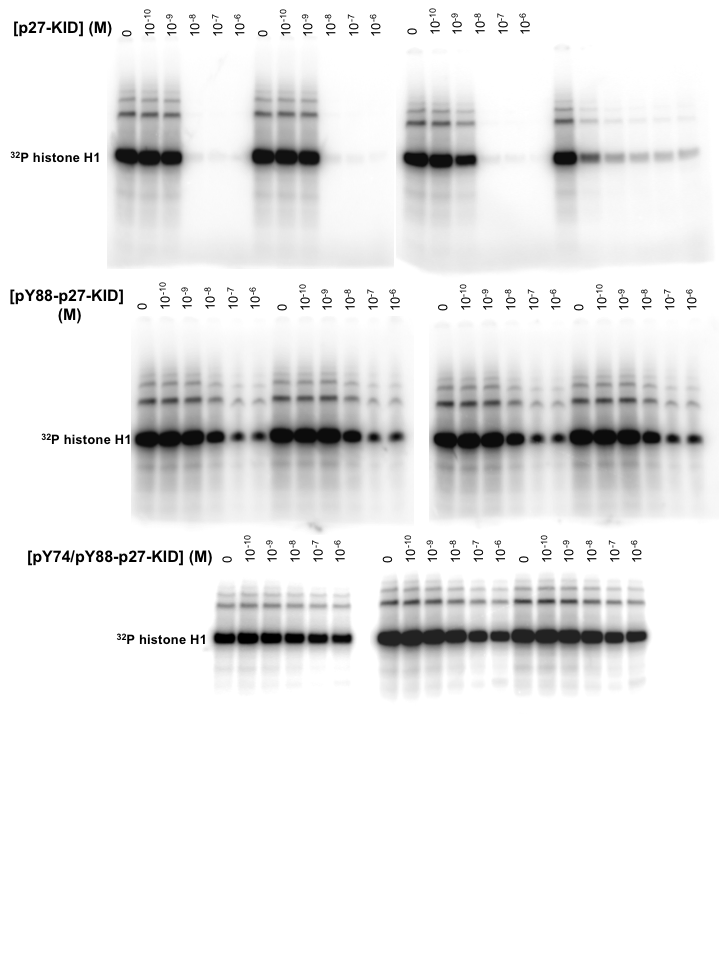
**

The unannotated lanes in the p27-KID samples were ran on the gel for a different experiment.

**Annotated complete phospho-image corresponding to Figure 2B.**

**
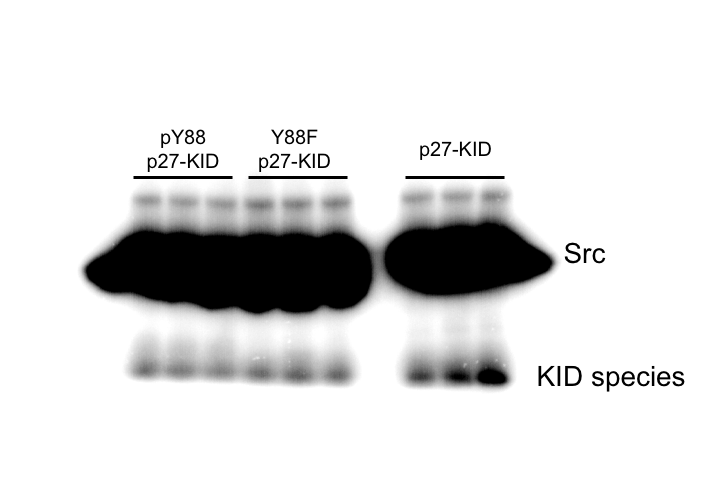
**

**
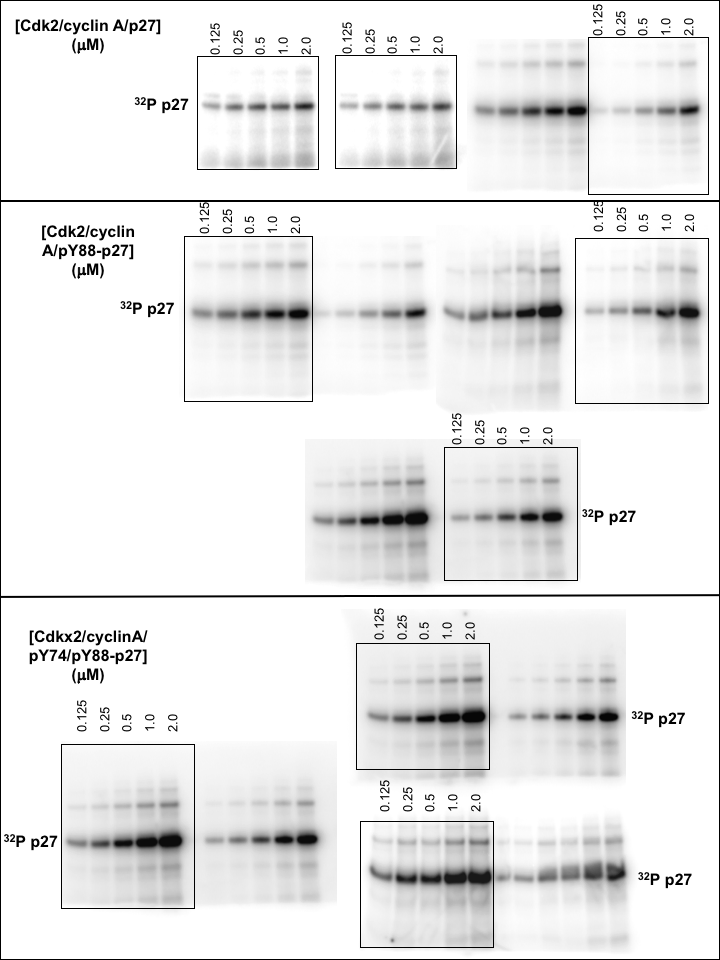
Annotated complete phospho-images corresponding to Figure 2C.**

**Annotated uncropped phospho-images corresponding to Figure 2D.**

**
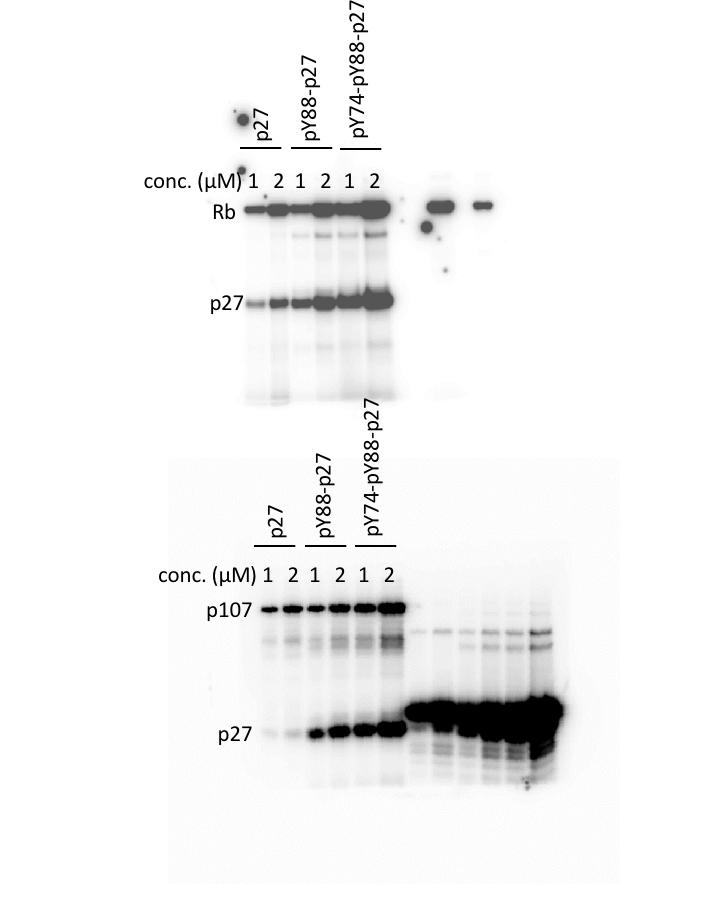
**

**Annotated uncropped phospho-image corresponding to Figure 2E.**

**
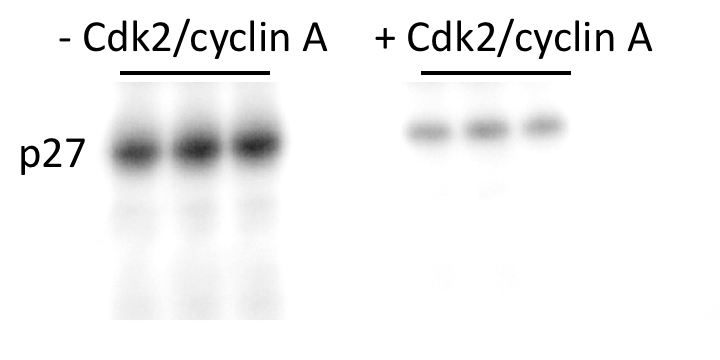
**

**Annotated uncropped phospho-image corresponding to Figure 2G**

**
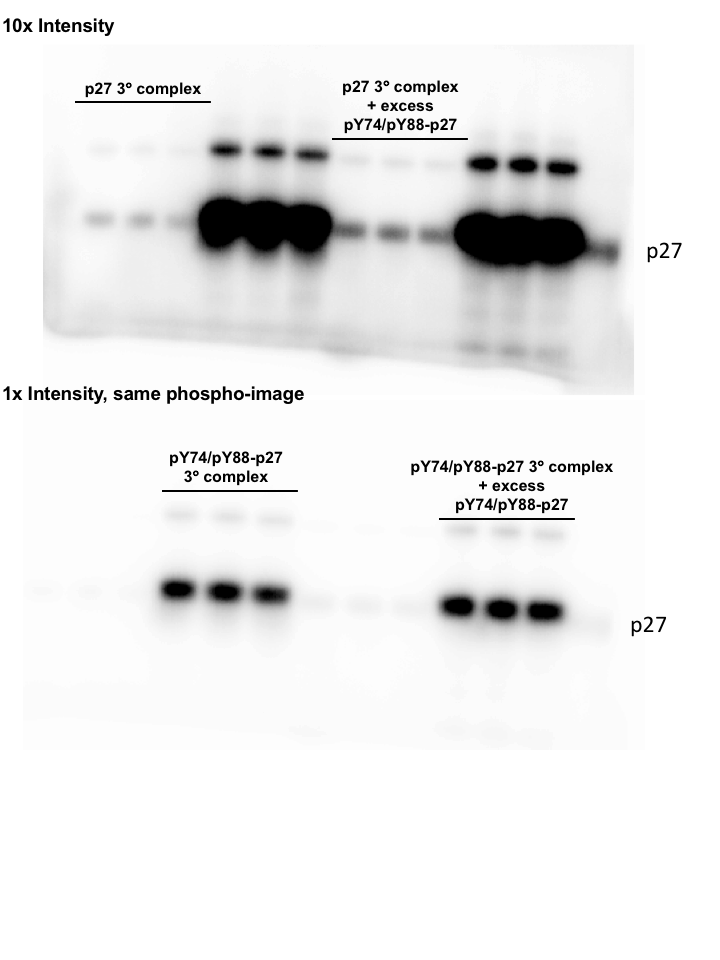
**
